# A Novel Approach Utilizing Domain Adversarial Neural Networks for the Detection and Classification of Selective Sweeps

**DOI:** 10.1002/advs.202304842

**Published:** 2024-02-02

**Authors:** Hui Song, Jinyu Chu, Wangjiao Li, Xinyun Li, Lingzhao Fang, Jianlin Han, Shuhong Zhao, Yunlong Ma

**Affiliations:** ^1^ Key Laboratory of Agricultural Animal Genetics Breeding, and Reproduction of the Ministry of Education & Key Laboratory of Swine Genetics and Breeding of the Ministry of Agriculture Huazhong Agricultural University Wuhan 430070 China; ^2^ Hubei Hongshan Laboratory Wuhan 430070 China; ^3^ Center for Quantitative Genetics and Genomics Aarhus University Aarhus 8000 Denmark; ^4^ CAAS‐ILRI Joint Laboratory on Livestock and Forage Genetic Resources Institute of Animal Science Chinese Academy of Agricultural Sciences (CAAS) Beijing 100193 China; ^5^ Livestock Genetics Program International Livestock Research Institute (ILRI) Nairobi 00100 Kenya; ^6^ Lingnan Modern Agricultural Science and Technology Guangdong Laboratory Guangzhou 510642 China

**Keywords:** domain adaptation, deep learning, genomics, robustness, selective sweep

## Abstract

The identification and classification of selective sweeps are of great significance for improving the understanding of biological evolution and exploring opportunities for precision medicine and genetic improvement. Here, a domain adaptation sweep detection and classification (DASDC) method is presented to balance the alignment of two domains and the classification performance through a domain‐adversarial neural network and its adversarial learning modules. DASDC effectively addresses the issue of mismatch between training data and real genomic data in deep learning models, leading to a significant improvement in its generalization capability, prediction robustness, and accuracy. The DASDC method demonstrates improved identification performance compared to existing methods and excels in classification performance, particularly in scenarios where there is a mismatch between application data and training data. The successful implementation of DASDC in real data of three distinct species highlights its potential as a useful tool for identifying crucial functional genes and investigating adaptive evolutionary mechanisms, particularly with the increasing availability of genomic data.

## Graphical abstract

This study investigates the risk of using simulated genomic data to train deep learning models for selective sweep detection. To address this, the study introduces a domain adaptation strategy and develops the Domain Adaptation Sweep Detection and Classification (DASDC) method. The results suggest that DASDC performs well in multispecies applications and demonstrates significant improvements in prediction robustness and accuracy.

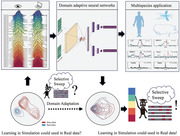

## 1 Introduction

Positive selection, an important driving force, plays a crucial role in biological evolution. Ideally, after a new beneficial mutation arises in the genome, positive selection will rapidly increase its frequency in the population. Because of linkage disequilibrium, the frequencies of variants flanking the beneficial mutation will also increase, a phenomenon that is known as genetic hitchhiking.^[^ ^1^ ^]^ Correspondingly, this genomic region with reduced genetic diversity is referred to as a selective sweep. Using appropriate statistical methods to identify selective sweeps facilitates the mapping of functional genes associated with phenotypic traits, which has significant implications in the medical field,^[^ ^2^, ^3^, ^4^ ^]^ plant and animal breeding,^[^ ^5^, ^6^ ^]^ as well as for understanding biological evolution.^[^ ^7^, ^8^ ^]^

Theoretically, selective sweeps can be further classified into hard and soft sweeps.^[^ ^9^, ^10^ ^]^ Hard sweep is defined as a single high‐frequency haplotype due to positive selection. In contrast to hard sweep, soft sweep is defined as more than one haplotype rising to moderate or high frequency due to selection. Previous studies found that soft sweeps are likely for alleles with a high fixation probability from the standing variation,^[^ ^9^ ^]^ while hard sweeps were rare in recent human evolution.^[^ ^11^ ^]^ Therefore, the soft sweeps may have relatively great implications for the adaptation of organisms to new environments.^[^ ^11^, ^12^ ^]^ However, a large proportion of the genomic features resulting from soft sweeps may not be readily discernible due to the broad range of initial frequencies of standing genetic variations and other influencing factors. Consequently, existing methods for detecting selective sweeps may not accurately capture them.^[^ ^13^, ^14^ ^]^

In general, selection will result in the following basic signals around a selected locus: (i) a shift of the allele frequency spectrum toward extreme frequencies; (ii) an excess of homozygous genotypes; and (iii) long haplotypes with high frequencies.^[^ ^15^, ^16^ ^]^ Accordingly, a series of user‐friendly and computationally efficient statistics, including Tajima's D,^[^ ^17^ ^]^ the integrated haplotype score (iHS)^[^ ^18^ ^]^ and ω statistic,^[^ ^19^ ^]^ have been developed to identify and characterize selection signatures across population genomic landscapes. However, these classical methods often have limited power to detect and classify selective sweeps due to their consideration of only certain characteristics and lack of robustness against genetic drift and population history, among other factors.^[^ ^20^ ^]^ To address these challenges, some methods integrating multiple characteristics have been developed based on classical statistics. These include statistical integration methods,^[^ ^5^, ^21^ ^]^ approximate Bayesian inference of selection events based on multiple characteristics,^[^ ^22^, ^23^ ^]^ and supervised machine learning‐based integration algorithms.^[^ ^24^, ^25^, ^26^ ^]^ These methods enhance the capture range of selective sweeps by integrating multiple signal characteristics, and the combination of these features reduces the effects of interference on detecting selective sweeps. Consequently, these approaches demonstrated superior performance compared with classical methods.^[^ ^27^, ^28^ ^]^ With the application of deep learning in life sciences and the enhancement of computing power, deep learning methods have gradually been applied to detect selective sweeps. In contrast to combining methods, deep learning not only possesses a stronger ability to express models but also has the capability to automatically extract features that are useful for tasks from data.^[^ ^29^ ^]^ Previous studies have demonstrated that deep learning‐based methods outperform the combined methods.^[^ ^30^, ^31^ ^]^

Currently, the primary challenge facing deep learning‐based methods is the scarcity of high‐quality labeled selective sweep data for model training.^[^ ^32^ ^]^ Therefore, existing deep learning‐based methods can only utilize simulated training datasets based on inferred population demographic history to train models and subsequently identify and classify selective sweeps in target populations.^[^ ^30^, ^33^, ^34^ ^]^ This significantly limits the applicability of these methods. Considering the challenge of accurately inferring population history, when there is a significant dissimilarity between the simulated data from the source domain used for model training and the real data from the target domain to be predicted by the model, features learned by the model in the source domain may not generalize well.^[^ ^35^ ^]^ Therefore, it is crucial to ensure the independence and identity of the distribution between simulated training data and real genomic data when utilizing these methods. However, real genomic data are much more complex than simulated data based solely on population inference history, making it difficult to meet this important premise.

Therefore, this study aims to find a suitable transformation function from domain adaptation that can approximate the feature distribution of the two domains after transformation and learn the relationship between the transformed features and labels under the source domain of known labels to ensure the generalization ability of the trained model under the target domain. Based on this idea, DASDC was developed by using a domain‐adversarial neural networks. It achieved this aim by introducing an adversarial learning module to align features by learning the invariant representations and using a fully connected layer to learn the mapping rules between the invariant representations and labels.^[^ ^36^ ^]^ To improve model stability, we employed a deep ensemble strategy for the development of our method.^[^ ^37^ ^]^ This study demonstrates that the novel approach exhibits superior generalizability and robustness. Furthermore, the DASDC software developed to support this method enhances its usability.

## 2 Results

### 2.1 Verification of the Basic Hypothesis for Detecting Selective Sweeps using Deep Learning‐Based Approaches

**Figure 1:**
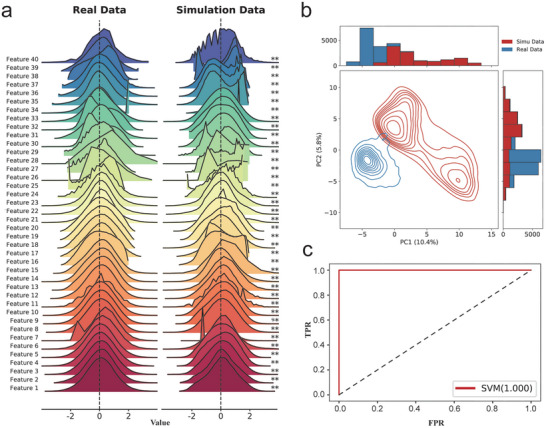
Empirical distribution difference test between simulated data and real data features. a) Probability distribution density between the 40 features of the real and simulated genomes of the CEU population, where ^*^ represents the significance of the difference obtained by the two‐sample K–S test (^*^: 0.01<*p* value ≤0.05, ^**^: *p* value ≤0.01). Similar results using the Mann–Whitney U test are shown in Figure S2 (Supporting Information). b) PCA results between simulated and real features of the genome with two principal components. The top and right bars represent the distributions of simulated features and real features for PC1 and PC2, respectively. c) ROC curve of the SVM for the discriminant results of simulated and real genome data.

Deep learning methods inherently assume that the simulated genomic data used for model training and the real genomic data follow an independent and identically distributed (i.i.d) pattern in the field of selective sweep detection. When this assumption does not hold, the model generalizability is compromised, meaning that the rules derived from the simulated data may not accurately extrapolate to real genomic data, thereby diminishing the effectiveness of the model on real datasets. To explore whether the simulated and real data followed the i.i.d. pattern, we generated 5 classes of datasets based on the inferred genetic parameters of the Northern and Western European Ancestry population (CEU). Then, we used 40 summary statistics as indicators to identify the mismatch between the distribution of simulated data and real data. The results indicated significant differences in the empirical distributions of the 40 summary statistics between the two domains (**Figure** **1**a; Table S1, Supporting Information). To investigate the difference in the multi‐feature joint distribution between the two datasets, principal component analysis (PCA) revealed distinct distribution patterns in the 2D space for both simulated and real data, once again confirming significant differences in their distributions (Figure 1b). We also observed a clear distinction between the simulated hard sweeps and the real genomic data (Figure S1, Supporting Information). Notably, Hernandez et al. also reported a paucity of hard sweeps in recent human evolution.^[^ ^11^ ^]^ These findings suggest that the features of hard sweeps based on simulated data are rarely observed in real genomic data, resulting in a significant discrepancy between the simulated hard sweeps and the real data.

Considering the limitations of PCA in capturing the nuances of distributional differences between features from two datasets due to its dimensionality constraints, we further employed a support vector machine (SVM) to assess the mismatch between the two datasets (for detail see Supporting Information). The results indicated that the SVM can easily distinguish between the two domains (AUC = 1.0) (Figure 1c). To eliminate the impact of model‐intrinsic differences on the findings, we performed control experiments wherein simulated and real data were randomly assigned positive and negative labels, respectively. As expected, these control experiments did not discern any significant difference when labels were randomized (AUC = 0.496 and AUC = 0.512) (Figure S3, Supporting Information). These tests indicated that there are significant differences in the characteristics between the simulated and real genomic data, and the models trained by the simulated dataset may suffer from generalization impairment when they are applied to the real data.

### 2.2 A Novel Approach for the Detection and Classification of Selective Sweeps

**Figure 2:**
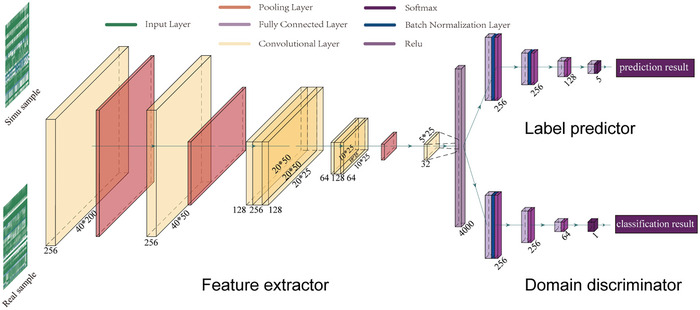
Diagram of the model architecture of DASDC. The green images represent the feature matrices of the simulated and real samples, orange represents the convolution layer, and red represents the pooling layer, which together constitute the feature extractor of the model. Light purple represents the fully connected layer, while blue and dark purple represent the batch normalization layer and ReLU, respectively. These three structures were then combined into predictors and discriminators.

Here, we proposed a novel approach defined as domain adaptation sweep detection and classification (DASDC), which utilizes an adversarial learning module to align features between source domain (simulated training dataset) and target domain (real dataset), thereby detecting and classifying selective sweeps by extracting invariant representations from two domain datasets (**Figure** **2**).

Different from the existing deep learning‐based sweep detection and classification methods that utilized larger (n*m, where n>1 and m>1) convolutional kernels to capture feature information,^[^ ^33^, ^38^, ^39^ ^]^ our study utilized 1*n and m*1 kernels to analyze the feature matrix. By harnessing 1*n convolutional kernels, we traced the distribution of statistical features along row vectors of the feature matrix, while m*1 kernels were employed to evaluate the relationships between different features at the same location encoded in the column vectors of the feature matrix (see Experimental Section for further details).

The performance of RNN and 1D CNN, based on sequence series information, was found to be inferior to that of 2D CNN which utilizes complete information from each region in the feature matrix (**Table** **1**), when comparing the enhancement of data utilization by our approach with other structures. The model performance was further improved by utilizing the 2D CNN with convolution kernels of size 1*n and m*1, as supported by the results of a one‐tailed *t*‐test: *p* = 0.004 for loss and *p* = 0.04 for accuracy. To investigate whether the improvement in model performance can be attributed to our designed first two convolutional layers, we conducted a concise ablation study by removing model components and evaluating their contributions to the overall performance (**Table** **2**, see Supporting Information for more details). The results showed that when the first two convolution layers were replaced or removed, the model's performance declined. This demonstrated that the structure used by DASDC can improve the predictive performance of the model.

**Table 1:** Comparison of the classification accuracy of the different feature extractors on the test dataset.

Feature Extractor	Loss	Accuracy [%]	Confidence
RNN	0.72 (0.03)	69.7% (0.02)	0.78 (0.04)
1D CNN	0.66 (0.02)	73.9% (0.01)	0.81 (0.02)
2D CNN	0.52 (0.02)	79.1% (0.01)	0.86 (0.02)
Our Model	0.49 (0.02)	80.23% (0.01)	0.86 (0.01)

The detailed computation of confidence was introduced in Supporting Information.

**Table 2:** Ablation experimental design and corresponding results.

Feature Extractor	Loss	Accuracy [%]	Confidence
Base model(DASDC)	0.49(0.02)	80.2% (0.01)	0.86 (0.01)
Remove the first two convolution layers	0.54(0.02)	77.8%(0.01)	0.84 (0.02)
Replace the first two layers of convolution	0.55(0.03)	77.4%(0.01)	0.84(0.02)

### 2.3 DASDC Shows Promising Generalization Capabilities

**Figure 3:**
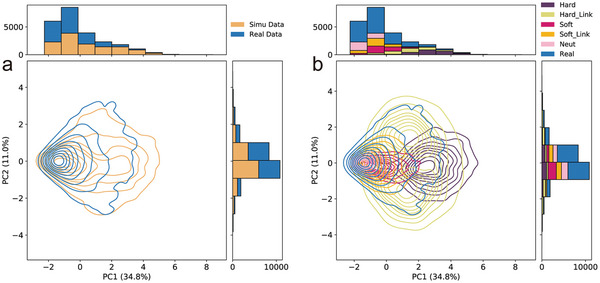
The feature extractor of DASDC reduced the distribution difference between simulation and real genome data. a) PCA results of simulated and real genome data passing through the feature extractor of DASDC. b) Visualization results of splitting the simulated data into five classes based on the following labels: hard sweep, linked hard sweep, soft sweep, linked soft sweep, and neutrality. The frequency histograms at the top and right of the graph represent the distributions of the sample values along PC1 and PC2, respectively.

To evaluate whether DASDC can effectively capture the invariant representations between the simulated and real data, the feature extractor of DASDC was used to process the two datasets independently, and the outputs were defined as deep learning features that were subjected to PCA. The results showed that the simulated and real data were clustered together by the first two principal components (PC) (**Figure** **3**a). Additionally, the PC1 and PC2 were significantly increased from 11.2% to 34.2% and from 6.3% to 11.1%, respectively, which was a strong indication of the effectiveness of the DASDC feature extractor in capturing the distinctions among data classes and then the invariant representations between the two datasets. Notably, the deep learning features in this study classified the simulated genome data into five classes based on labels: hard sweep, linked hard sweep, soft sweep, linked soft sweep, and neutrality. The PCA visualization showed clear differentiation between the hard, linked hard, and soft sweeps, while some distinctions were also found between the linked soft and neutral classes (Figure 3b). This result further proved that the extracted invariant representations contained structural information between the different selective sweep classes. These findings demonstrated the capacity of our DASDC feature extractor to alleviate the difference in feature distribution and to capture the characteristics of selective sweep classes, which was an efficient approach for resolving impaired generalization when the models were used for real data.

### 2.4 The Performance of DASDC in Mismatch Scenarios

**Figure 4:**
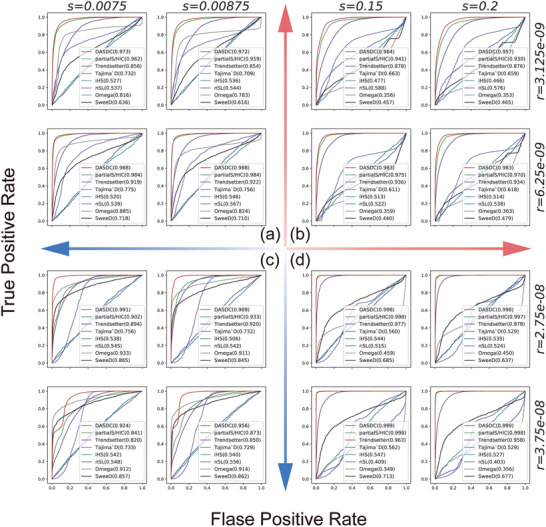
Comparison of methods in 16 scenarios with mismatches. The selection coefficient parameters and the recombination rate parameters are shown at the top and right of the figure, respectively. Different methods correspond to different curves, with DASDC in red, partialS/HIC in light green, Trendsetter in purple, iHS in light blue, nSL in dark blue, ω in gray, and SweeD in black.

Distributional differences between the simulated training data and real data were inevitable due to the impossibility of knowing the true population demographic parameters. To assess the potential impacts of this issue, we focused on two population genetic parameters, recombination rates and selection coefficients, and simulated 16 scenarios in which the two parameters were mismatched from the training datasets for DASDC performance evaluation and comparison among methods (see Experimental Section for further details). According to the mismatches in population genetic parameters, the 16 scenarios were divided into four categories: (i) lower recombination rate and selection intensity; (ii) lower recombination rate but higher selection intensity; (iii) higher recombination rate but lower selection intensity; and (iv) higher recombination rate and selection intensity (**Figure** **4**).

In categories (i) and (iv), DASDC is slightly better than partialS/HIC and better than Trendsetter and the traditional methods (Figure 4a,d). This may be because in the mismatched scenarios mentioned above, the degree of mismatch from recombination and selection cancels each other out, resulting in a selection signal that closely resembles that of the source domain. This is according to s/[4*Ne*r*log(Ne*s)], an approximate function for computing the size of the hard sweep footprint, where s represents the selection coefficient, Ne represents the effective population size, and r represents the recombination rate.^[^ ^40^ ^]^ The results of PCA support this inference that iv < i < ii < iii in the degree of mismatch (Figure S4, Supporting Information). Therefore, each method performs well. In category (ii), DASDC is better than partialS/HIC, Trendsetter and traditional methods (Figure 4b). In this category, the change in the direction of the two parameters leads to further mismatch between the selected features and those of the source domain, but low recombination does not cause feature confusion. Therefore, in this category, the performance of each method is reduced compared with that in the (i) and (iv) categories, but the advantages of DASDC over the other methods are initially highlighted. In category (iii), the duration of selection events was longer due to the low selection coefficient, which increased the number of recombination events in the genome and thus resulted in a dilution of selection features. Therefore, these scenarios had the greatest impact on the features, causing the performances of partialS/HIC and Trendsetter to decline again. However, DASDC exhibits only a slight degradation in performance, making it the best performing method with more prominent advantages over the other methods (Figure 4c). This suggests that DASDC has obvious robustness advantages over the other methods, making it more applicable to real genome data that exhibits mismatches compared to its simulated training data.

**Figure 5:**
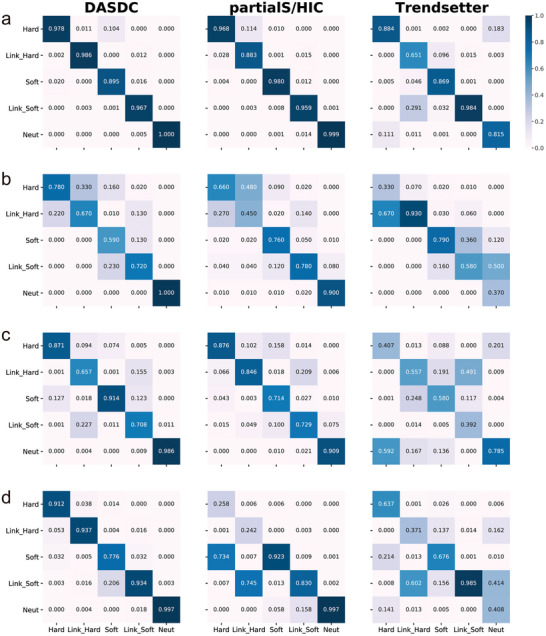
Error analysis in various mismatch scenarios. Heatmap of the confusion matrices for the three methods applied to four scenarios with mismatches in the training data. The distinction among these four scenarios lies in the variation of recombination rates and selection coefficients used: a) high recombination rate (r = 3.75e‐08) and high selection coefficient (s = 0.2), b) low recombination rate (r = 3.125e‐09) and high selection coefficient (s = 0.2), c) low recombination rate (r = 3.125e‐09) and low selection coefficient (s = 0.0075), and d) high recombination rate (r = 2.75e‐08) and low selection coefficient (s = 0.0075). The horizontal coordinate represents the true label and the vertical coordinate represents the predicted label.

To further evaluate the classification performance of DASDC in the scenarios with mismatched population genetic parameters, confusion matrices were drawn for DASDC, partialS/HIC and Trendsetter for these 16 scenarios (**Figure** **5**; Figure S5, Supporting Information). We divided the misjudgment scenarios of the model into three categories: (i) misjudgments between the hard sweep and soft sweep categories; (ii) misjudgments between the selection categories and their corresponding linkage categories; and (iii) misjudgments between the neutral and linked soft sweep categories. Out of these 16 scenarios, four were selected as representatives to demonstrate the classification performance (Figure 5). In the scenario with a high recombination rate and high selection intensity, all three models demonstrated excellent classification performance with virtually no misjudgments (Figure 5a). In the scenario with a low recombination rate and high selection intensity, although all methods showed increased misjudgments between selection categories and corresponding linkage categories, the accuracy of DASDC classification improved by an average of 4.2% compared to partialS/HIC. Additionally, there were no misjudgments between neutral and linked soft sweep categories (Figure 5b). In the scenario with a low recombination rate and high selection intensity, the accuracy of DASDC classification only improved by an average of 1.2% compared to that of partialS/HIC, both of which were better than that of Trendsetter (Figure 5c). In the scenario with a high recombination rate and low selection intensity, all three methods exhibited all three misjudgments. However, DASDC showed a clear advantage in classification compared to the other methods. PartialS/HIC lost its ability to distinguish between hard and soft sweep categories and tended to classify hard sweep and its linkage categories as soft sweep and its linkage categories. The confusion among Trendsetters is even worse, with various misjudgments (Figure 5d). Therefore, although the DASDC method also had misjudgments in various scenarios, compared to other methods, the resolution of the DASDC method was improved to a certain extent, and its classification performance was excellent in the most serious mismatched scenario with a high recombination rate and low selection intensity.

### 2.5 The Performance of DASDC in Ideal Scenarios

In this study, we conducted a performance evaluation of DASDC and several methods under an ideal scenario where both the target domain (real data) and source domain (simulated training data) were uniform. The DASDC, partialS/HIC, and Trendsetter methods were independently trained 10 times, and the optimal model was selected for performance evaluation. As expected, DASDC and partialS/HIC performed better, while Trendsetter performed worse than them but better than the traditional methods. We noted that partialS/HIC was slightly better than DASDC for hard sweep detection, but the DASDC method had a greater advantage in soft sweep detection (Figure S6, Supporting Information).

Since Trendsetter, DASDC, and partialS/HIC default to using 6, 40, and 89 summary statistics, respectively, within their algorithms, the performance differences may be attributed to the utilization of different feature statistics. Therefore, we further investigated the performances of DASDC, partialS/HIC, and Trendsetter using identical feature statistics. Under a constant demographic model, all methods demonstrated strong performances, with DASDC (AUC = 0.997) slightly outperforming partialS/HIC (AUC = 0.995) and Trendsetter (AUC = 0.994) (Figure S7, Supporting Information). This result indicates that all three methods possess strong capabilities for the classification and detection of selective sweeps. However, in the CEU demographic, DASDC (AUC = 0.967) outperforms partialS/HIC (AUC = 0.941) and Trendsetter (AUC = 0.950) to a greater extent (Figure S8, Supporting Information), indicating that the structural design of our model enhances its robustness to variations in population demographics compared to the other methods. Moreover, Trendsetter showed a significant improvement in performance when utilizing the feature set designed for DASDC (from an AUC of 0.88 with its default 6 features), whereas partialS/HIC experienced a decrease in performance (from an AUC of 0.965 with its default 89 features), suggesting that feature choice has a substantial impact on method performance.

**Figure 6:**
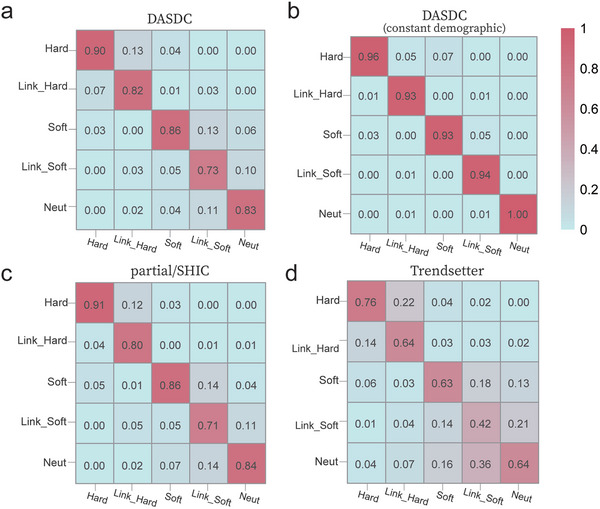
Error analysis and performance evaluation of DASDC in ideal scenarios. a) Performance of DASDC in the CEU demographic scenario. b) Performance of DASDC in the constant demographic scenario. c,d) Performance of partialS/HIC and Trendsetter in the CEU demographic scenario, respectively. The horizontal coordinate represents the true label and the vertical coordinate represents the predicted label.

In these ideal scenarios, we also assessed the classification performance of the DASDC method. The confusion matrix showed that there were three main sources of classification misjudgment: (i) approximately 13% of the linked hard sweeps were misjudged as hard sweeps, and 7% of the hard sweeps were misjudged as linked hard sweeps; (ii) approximately 13% of the linked soft sweeps were misjudged as soft sweeps, and 5% of the soft sweeps were misjudged as linked soft sweeps; and (iii) approximately 11% of the linked soft sweeps and 4% of the linked soft sweeps were misjudged as neutral, and approximately 10% and 6% of the neutral category was misjudged as linked soft sweeps and soft sweeps, respectively (**Figure** **6**a). There was little confusion between hard and soft sweeps in our model, and almost no confusion among hard sweeps, linked soft sweeps, and the neutral category. This indicates that our model had a high accuracy in detecting hard sweeps.

Subsequently, we explored the reasons for misjudgment, and we inferred that the misjudgments may have been caused by the interference of population history. To validate our hypothesis, we assessed the performance of DASDC on a constant demographic dataset. The results indicated that the degree of misjudgment in all three categories was reduced, and no misjudgments occurred between the selection and neutral categories (Figure 6b). This validates our hypothesis that the model confuses the characteristics of soft sweeps and neutrality in the presence of interference from population history, resulting in a decrease in the precise localization ability of detection. The misjudgment between soft and hard sweeps may have been caused by the soft shoulder effect, i.e., the neutral region adjacent to the hard sweeps might show characteristics similar to those of the soft sweeps because of the interference of mutation, recombination, and other factors.^[^ ^41^ ^]^

Finally, we compared the classification performances of DASDC, partialS/HIC and Trendsetter in ideal scenarios. We found that DASDC was slightly better than partialS/HIC and better than Trendsetter (Figure 6a,c,d). The categories of misjudgments were the same for all three methods, encompassing all three types of misjudgment as described above. Among them, DASDC classified the linkage and selection categories slightly better than partialS/HIC (Figure 6a,c), possibly due to the use of sequence series information. Trendsetter made significant errors in distinguishing between selection and linkage categories, which may be attributed to the fact that this method initially aimed for three classifications but employed only 6 statistics for feature engineering construction.^[^ ^25^ ^]^

### 2.6 The Robustness of DASDC

In the analysis of selective sweeps, it is challenging to distinguish missing genomic regions resulting from sequencing technology or subsequent quality control from the reduction in genomic polymorphisms caused by selection. Here, we assessed the impact of missing genomic regions on the model robustness and found that the classification accuracy of the model was moderately affected, particularly in the neutral scenario, where 59.3% of the neutral classes were misclassified as soft linkage sweeps, 29.6% as soft sweeps, and only 15% were correctly predicted (Figure S9a, Supporting Information). In addition, the discriminative ability between the sweeps and linkage sweeps declined. Nevertheless, the model exhibited robust detection capabilities for both hard and soft sweeps. Note that DASDC displayed robustness against missing genomic regions when 20% of the missing data were considered as the source domain (Figure S9b, Supporting Information). This suggests that prior consideration can effectively mitigate the influence of missing genomic regions.

Background selection, a prevalent phenomenon across the genome,^[^ ^42^ ^]^ can give rise to the appearance of neutral features that are subjected to positive selection in low‐recombination regions. Thus, we evaluated the effect of this factor on our method and found that DASDC exhibited good robustness across the four background selection scenarios (Figure S10, Supporting Information). Similarly, recombination rate heterogeneity is also a potential interfering factor in the detection of positive selection.^[^ ^43^ ^]^ Our evaluation showed a significant decline in the model performance in this scenario (Figure S11a, Supporting Information). Specifically, hard sweeps were exclusively misclassified as soft or neutral classes, and the identification rate for soft sweeps was only 29% (Figure S11b, Supporting Information). Nevertheless, the performance improved significantly when the training data comprised a combination of 50% recombination heterogeneity and 50% homogeneity. In this scenario, the accuracies for detecting neutrality, hard sweep, and soft sweep were 98%, 76%, and 71%, respectively (Figure S11c, Supporting Information). Further trials using training sets composed entirely of recombination heterogeneity data yielded only a slight improvement in performance (Figure S11a,d, Supporting Information), suggesting that incorporating a proportion of the recombination rate heterogeneity during training was sufficient to enhance the robustness of the DASDC in such scenarios.

In addition to the recombination rate heterogeneity, overcoming the confusion between low recombination rates and selective sweeps is a challenge in methodological research. Here, we assessed DASDC in four scenarios in which the recombination rate was reduced relative to the source domain by 5‐, 10‐, 50‐, and 100‐fold. It was found that even when the recombination rate was reduced 10‐fold, the DASDC method maintained good detection and classification performance. Although the performance of DASDC was reduced and led to false negatives (with almost all soft sweeps predicted as soft linkage sweeps) with a 100‐fold reduction in the recombination rate, the method maintained good control over false positives (with only 6% of the neutral class predicted as sweep classes) (Figure S12, Supporting Information).

Finally, we explored the impact of category imbalance, which is a potential issue particularly relevant to real data with an undetermined number of swept regions. The results suggested that when there was no covariance shift, category imbalance did not affect the classification accuracy of the model (Figure S13a–c, Supporting Information). However, when a covariance shift between the domains was present, a category imbalance could lead to a further decline in the model performance (Figure S13d,e, Supporting Information). Considering that a covariance shift is often unavoidable in model applications, correcting category imbalance is worth exploring.

### 2.7 The Assessment Results of Feature Importance in DASDC

To understand the important features of the input matrices for prediction, saliency maps were highlighted in both mismatched and ideal scenarios. In the ideal scenario, the hard sweeps category showed a pronounced focus on the fourth and fifth moments of the SFS, *K* and haplotype heterozygosity, while the soft sweeps category paid more attention to H12, H123, and H2/H1 (Figure S14, Supporting Information). In the mismatched scenario, the model has broadened to encompass a more diverse range of features, including not only certain HAF and φ characteristics but also various SFS attributes and a wider array of features (Figure S15, Supporting Information). These results suggest that the model will focus on different features in various scenarios. This may indicate that more features are useful for the model to identify the invariant representations in scenarios where mismatch exists. In addition, we noted that the features of interest for selection and linkage classes were aligned in both scenarios but differed in the mapping location. This is consistent with our expectations that locational information is important for distinguishing between selection and linkage classes.

### 2.8 The Application of DASDC Approach in Real Genomic Data From Different Species

**Figure 7:**
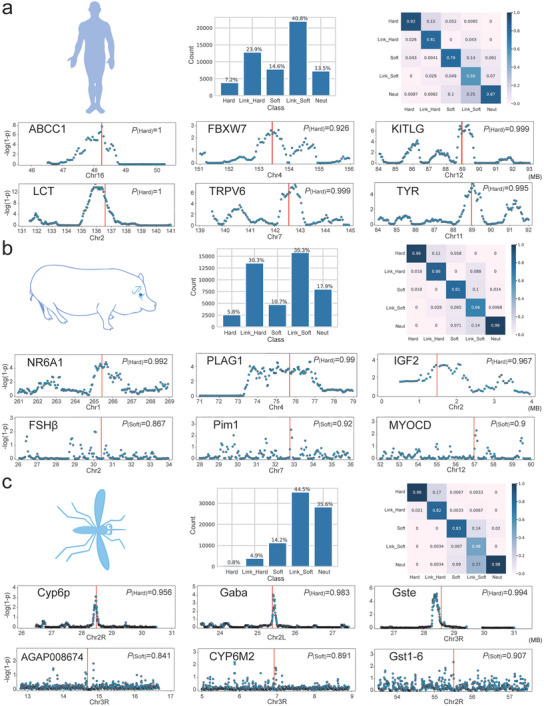
Visualization results of detecting and classifying Selective Sweeps in real data and highlighting partially established selective sweeps. a) In CEU population of humans, b) in Large White pigs of domestic animals, and (c) in BFS Anopheles of insects.

To explore the cross‐species applicability and generalizability of our DASDC approach to real data, we conducted selective sweep detection on three distinct populations: the CEU population of humans,^[^ ^44^ ^]^ the Large White (LW) pig population of domestic animals, and the Anopheles of Burkina Faso (BFS) population of insects.^[^ ^45^ ^]^ As shown in Figure S16 (Supporting Information), the models were trained based on inferred specific population genetic parameters. Distributions of the three real genome datasets on the domain discriminator were concentrated to the left at 0.5. This indicated effective alignment performance, as corroborated by the performance of DASDC in the source domain depicted in **Figure** **7**, suggesting its proficient classification capabilities. Additionally, a basic statistical analysis of the DASDC prediction results revealed a distribution feature of the output probabilities that aligned with the misjudgment analysis under mismatched scenarios (Figure S17, Supporting Information), indicating that the model's performance remained within our expected scope.

Subsequently, we detected and classified selective sweeps in the whole genomes of the three populations. Statistical analysis of the classification results (Figure 7) revealed that in the genomes of the three species, soft sweeps significantly outnumbered hard sweeps, which is consistent with previous findings.^[^ ^12^, ^33^ ^]^ Moreover, mosquitoes demonstrated the highest proportion of soft sweeps, followed by humans, while pigs exhibited the lowest. This may be attributable to the frequent environmental changes faced by mosquitoes due to anti‐mosquito measures such as insecticides, which create ample conditions for soft selection.

#### 2.8.1 Application to Human Population

For the CEU population, established selective sweeps such as the *LCT* gene related to lactose tolerance,^[^ ^46^ ^]^ the *ABCC1* gene related to earwax and sweat production,^[^ ^47^ ^]^ and the *TYR*, *TRPV6*, and *KITLG* genes related to pigmentation^[^ ^48^, ^49^ ^]^ were considered as the gold standard for evaluating the performance of DASDC applied to human populations. The identification of these established selective sweeps demonstrated the potential of our method (Figure 7a). Note that both the S/HIC and Trendsetter methods identified the *LCT* gene as a soft sweep, while the DASDC method classified it as a hard sweep. In addition to the established selective sweeps, by annotating the functions of the candidate regions for selective sweeps, we identified other genes under selection, such as *KAT6B*, which is involved in brain development, and the tumor suppressor genes *HINT1* and *MDH1B*, which are related to glucose metabolism (Table S2, Supporting Information).

#### 2.8.2 Application to Domestic Animals

For the LW population, genes such as *NR6A1* and *PLAG1* associated with body length,^[^ ^50^ ^]^ *IGF2* associated with growth^[^ ^51^ ^]^ and *FSH𝛽* associated with litter size^[^ ^52^ ^]^ were also successfully identified in this study (Figure 7b). This indicated the effectiveness of DASDC on domestic animals with uncertain demographic histories. In addition, bioinformatics analysis revealed that genes annotated in the soft sweep regions were more likely to contribute to the fitness of LW pigs, such as *Pim2*, which promotes myocardial regeneration, and *MYOCD*, which mitigates myocardial damage, compared to those annotated in hard sweep regions (Table S3, Supporting Information).^[^ ^53^, ^54^ ^]^ However, it should be noted that both hard and soft sweeps play important roles in the adaptive evolution of pigs. In addition, we also discovered several genes that have not been reported for their functions in pigs, including *GJA1*, which encodes a protein that plays a crucial role in the synchronized contraction of the heart and embryonic development, and *TUSC3*, which encodes a protein associated with embryonic development.

#### 2.8.3 Application to Insect Populations

For the BFS population, we used genes known for their functions related to *Anopheles gambiae* drug resistance as the gold standard. These include *Cyp6p*, *CYP6M2*, *Gst1‐6* and *AGAP008674*, which are associated with pyrethroid resistance^[^ ^55^ ^]^; *Gste2*, which is related to DDT resistance^[^ ^56^ ^]^; and *Gaba*, which is associated with drug resistance.^[^ ^45^ ^]^ These results demonstrated the effectiveness of DASDC in insects (Figure 7c). The successful detection of these genes demonstrated the multispecies applicability of our method. Furthermore, we annotated the candidate sweep region and detected numerous candidate genes related to lipid and biological metabolism that are beneficial for blood feeding and drug resistance in *Anopheles gambiae* (Table S4, Supporting Information).

## 3 Discussion

In this study, *LCT* gene was classified as a hard sweep, while both S/HIC and Trendsetter methods identified *LCT* as a soft sweep. We considered two possibilities: (i) DASDC corrects potential differences between simulated and real genomic features, thereby reducing the impact of distributional differences and obtaining more accurate classification results, and (ii) there may be significant differences in characteristics between the simulated genome and the real genomes, with *LCT* genes undergoing strong natural selection that could exhibit features similar to those observed during a hard sweep in simulated genome data. However, the accuracy of the human population demographic and population genetic parameter inference, combined with the robustness of the DASDC method in mismatched data, led us to lean toward *LCT* being subjected to a hard sweep in the CEU population. Note that DeGiorgio and Szpiech employed a composite likelihood method based on the spatial distribution of the haplotype frequency spectrum to identify *LCT* as a hard sweep.^[^ ^57^ ^]^ Furthermore, the estimated occurrence time of *LCT*, as discussed by Peter et al.,^[^ ^58^ ^]^ suggests that if it originated from a neutral mutation, the allele may have been produced > 480 kyr ago. However, the allele was not found in Eastern European samples dating back approximately 7000 years. Alternatively, if *LCT* arose from a de novo mutation, the allele could have emerged approximately 5000 years ago, with dairy farming dating back to 11–12 kyr ago, providing optimal environmental conditions for selection. Overall, it is more likely to be subjected to a hard sweep.

With the increase in genomic data and advancements in computational power, numerous machine learning‐based and deep learning‐based methods for detecting selective sweeps have been developed. Compared with traditional methods, these methods exhibit superior performance because they account for population‐specific genetic parameters and other factors. However, these approaches also have certain limitations. This study illustrates another key factor restricting the performance of deep learning‐based methods in addition to their high computational cost: satisfying their assumptions is difficult. There are still significant differences between the generated pseudo data and real genomic data, even if the population demographic parameters can be more accurately inferred for data simulation. Therefore, deep learning‐based methods cannot avoid some deviations when used on real genomes. To address this challenge, there are two primary solutions so far. One solution involves simulating data that closely match the real genomic sequences for model training. In this way, methods such as PG‐GAN implement a generative learning strategy, allowing models to produce simulated genomes that bear a close resemblance to actual genomic data.^[^ ^59^, ^60^ ^]^ The second solution involves constructing features on simulated and real genomes with distributions as similar as possible for model training, ensuring that the model (eg. Flex‐sweep) can generalize to real data.^[^ ^61^ ^]^ Here, we adopted a new approach by utilizing a domain adversarial neural network to facilitate the extraction of invariant representations that are useful for classification tasks from both real and simulated data. Notably, our idea does not conflict with the above solution. It can be considered an exploration of this issue from three different aspects: original data, feature engineering, and model structure. Perhaps the combination of these three ideas may further improve the performance and robustness of the deep learning‐based methods. In addition, the domain adversarial neural network adopted by DASDC can be regarded as adding a regularization in the form of an adversarial learning module on the basis of a deep neural network for extracting invariant representations that are useful for classification tasks, and the regularization parameters can be optimized by fine‐tuning the structure of the adversarial learning module or hyperparameter λ. Therefore, this idea can be extended to existing deep learning‐based methods. Furthermore, we note that Mo and Siepel have employed a similar strategy in population genetic inference, which has also been proven effective in this strategy.^[^ ^62^ ^]^

However, with an increase in the difference in feature distribution, although DASDC performed better than Trendsetter and partialS/HIC, its performance still inevitably declined. In addition, under scenarios with large differences in feature distribution, DASDC performed worse than some traditional statistical methods. Here, we elaborate upon this phenomenon in detail. The upper and lower bounds of the error when the model is used in the target domain can be expressed as^[^ ^63^ ^]^:

(1) $$\begin{array}{c} \varepsilon_{T}\left(h\right)\leq {\hat{\varepsilon }}_{S}\left(h\right)+d_{\tilde{H}}\left({\hat{D}}_{S},{\hat{D}}_{T}\right)+2{\mathrm{Rad}}_{S}\left(H\right)+4{\mathrm{Rad}}_{S}\left(\tilde{H}\right)\\ \;\;\;\;+min\left\{E_{D_{S}}\left[\left|f_{S}-f_{T}\right|\right],E_{D_{T}}\left[\left|f_{S}-f_{T}\right|\right]\right\}\\ \;\;\;\;+O\left(\sqrt{\log\left(1/\delta \right)/n}\right), \end{array}$$

(2) $$\varepsilon_{T}\left(h\right)+\varepsilon_{S}\left(h\right)\geq \frac{1}{2}{\left(d_{\mathrm{JS}}\left(D_{S}^{Y},D_{T}^{Y}\right)-d_{\mathrm{JS}}\left(D_{S}^{Z},D_{T}^{Z}\right)\right)}^{2}$$

where ε_*T*_(*h*) is the error of the target domain, $d_{\tilde{H}}\left({\hat{D}}_{S},{\hat{D}}_{T}\right)$ is the distance between the source domain and target domain distributions; ${\mathrm{Rad}}_{S}\left(H\right)$ and ${\mathrm{Rad}}_{S}\left(\tilde{H}\right)$ are the Rademacher complexities of the hypothesis spaces H and $\tilde{H}$, respectively. They depend on the complexity of the model and the training dataset, so these two values are similar in many scenarios. $E_{D_{S}}\left[|f_{S}-f_{T}|\right]$ represents the expectation of differences using the source domain label function *f_S_* and the source domain label function *f_T_* in the source domain $D_{S}$, $O(\sqrt{\log(1/\delta )/n})$ is the error caused by finite samples, $d_{\mathrm{JS}}\left(D_{S}^{Y},D_{T}^{Y}\right)$ is the distance quantization between the marginal label distributions of source and target domains, and $d_{\mathrm{JS}}\left(D_{S}^{Z},D_{T}^{Z}\right)$ is the distance quantization between the deep features of the source and target domains.

For the upper error bound, because deep learning‐based methods commonly assume that the model can learn prediction rules from simulated data that apply to real data, that is, *f_S_* = *f_T_* , the error limit depends on the error of the model in the source domain and the distance between the two domain distributions, as well as the complexity of the hypothesis spaces H and $\tilde{H}$. In this regard, previous deep learning methods can only reduce the error of the first term, while DASDC goes a step further by reducing the error of the second term through extracting invariant representations based on this. This explains why DASDC exhibits better performance in many scenarios. However, the error analysis revealed that significant differences in feature distribution between the two domains resulted in concept shift, which altered the relationship between samples and labels (that is, *f_S_* ≠ *f_T_*). Regarding the error upper bound, concept drift increases the error term $min\{E_{D_{S}}\left[|f_{S}-f_{T}|\right],E_{D_{T}}\left[|f_{S}-f_{T}|\right]\}$. To some extent, this reveals why the performance of deep learning‐based methods deteriorates when the distribution difference is large. Meanwhile, it also indicates that when DASDC is used for real data prediction, the feature difference between the simulated and real data should not be too large to avoid the degradation of model prediction results caused by concept drift.

For the lower error bound, the error limit depends on $d_{\mathrm{JS}}\left(D_{S}^{Y},D_{T}^{Y}\right)$ and $d_{\mathrm{JS}}\left(D_{S}^{Z},D_{T}^{Z}\right)$. To narrow the lower bound, both Trendsetter and partialS/HIC aim to minimize the difference between the second and first terms. However, obtaining the first term is difficult due to the unavailability of real labels, which makes it challenging to determine a criterion for training the model and reducing the lower bound. For DASDC, we aimed to minimize the error in the second term during the training process. This provided a clearer criterion for narrowing the lower bound, with the aim of minimizing the error in the first term as much as possible. This also indicates another way to improve the generalization of deep learning‐based methods: making prior assumptions or correcting the label distribution of the source domain to align it as closely as possible with that of the target domain.

## 4 Conclusion

Research indicates that achieving independent and identical distributions of source domain training data and target domain data is a challenging task for selective sweep detection methods based on deep learning. Here, we developed a DASDC method by using a domain adversarial network, which was used to align features by learning the invariant representations between simulated and real data, and the model classification function was optimized by using the labels of the simulated data. This study presents a novel solution to the generalization issue of selective sweep detection methods by utilizing a deep learning model. Finally, a DASDC software was developed, which integrates the simulation functionalities of discoal and provides users with an easy‐to‐use tool for selective sweep detection and classification.

## 5 Experimental Section

### Model Architectures—Choice of Statistics for Extracting the Selective Sweep Feature

In this study, a total of 40 summary statistics were selected from three perspectives to extract the features of selective sweeps (Table S1, Supporting Information).

- The basic features used to distinguish between selection and neutral classes: the statistics of ΔAF, π, *watterson*′θ, *H*_θ_, and *Fay* *Wu*′*H* were used to describe the genomic polymorphism; the 1st to 5th statistical moments and entropy were used to describe the characteristics of the site frequency spectrum; the number of haplotype categories (N), haplotype heterozygosity, iHS, *nS*_L_, and Δi*HH* were used to describe the haplotype features containing the decay degree of linkage disequilibrium and the distribution of the site frequency spectrum.
- Identifying the favored mutation under selection used to distinguish between selection and linkage classes: the ability of the model to distinguish the linkage class and selection class can be improved by providing the features of mutation favored by selection. Therefore, we choose the SAFE score and its derivative statistics Φ and 𝜅 to provide this information.^[^ ^64^ ^]^
- The features used to distinguish the hard sweeps from the soft sweeps include H1, H12, H123, H2/H1 and HAF scores.^[^ ^65^, ^66^ ^]^

### Model Architectures—Genomic Selective Sweep Feature Engineering Construction

To enable the model to better utilize the extracted features based on the above summary statistics, the following rules were formulated to convert each sample into a corresponding feature matrix (Figure S18, Supporting Information). For simulation data, each case was treated as a sample. For real data, *w* was defined as the size of the sliding window along the genome, and s was defined as the step length. Based on the w and s values, the genome was divided into multiple genomic fragments. Each fragment was considered as an independent sample for feature engineering. The process was as follows:

- Feature Window: Each sample was divided into n windows, with each window containing 2k + 1 SNPs, where 1 represents the central SNP, and 2k represents the SNPs extending k upstream and downstream of the central SNP. The process is achieved by uniformly extracting n central SNPs from the samples (Figure S18a, Supporting Information). The positional information of each window was recorded by calculating the average physical position of the SNPs within it. If the number of SNPs in each sample is < 2*k + n, the sample was excluded.
- Feature Computation: We calculate 40 summary statistics for each of these n windows, resulting in a matrix of dimensions 40*n. Each row represents the values of a specific summary statistics feature across n windows for a sample, and each column represents the values of the 40 features in a specific window (Figure S18b, Supporting Information). The detailed calculation process for each statistic is described in Supporting Information.
- Positional Encoding: Each sample was evenly divided into *m* intervals based on the length of genomic fragment. Each statistical value was then assigned to its corresponding interval based on the physical position information of the window, and the average value for each interval was calculated (Figure S18c,d, Supporting Information). If an interval did not contain any statistical values, it was filled with zeros to indicate a lack of variation in that genomic region.

Here, sliding windows with a fixed number of SNPs were employed to capture genomic features characterized by reduced polymorphism due to selection, as selection regions typically exhibit larger genomic spans than neutral regions. However, the results in each column of the 40*n matrix obtained in (ii) only reflect the ordinal information between windows, without accurately representing the positional information of features (Figure S18a, Supporting Information). This may compromise the model's performance in distinguishing between selections and linkages, particularly when there is a significant mismatch between ordinal and positional information. Hence, the features computed based on windows with a fixed number of SNPs were finally mapped to their respective positions in the genome using Operation (iii). Through the application of this calculation rule, we successfully quantized the sample (the genomic fragment) into a feature matrix *M* of size 40 * *m*. *M_ij_* represents the feature values of the *i_th_* statistic at the genomic fragment of [$L\;*\;\frac{j-1}{m}$, $L\;*\;\frac{j}{m}$]. This can be interpreted as quantification of the *i_th_* feature of the *j_th_* genomic fragment. Here, *L* is defined as the length of the chromosome or simulated genomic fragment. In the DASDC software, the default parameters of the above process were set as follows: *n* = 200, *k* = 25, *m* = 200, *w* = 1000,000 bp, and *s* = 50,000 bp. Finally, the feature vectors of the training, validation, test, and target domain datasets were max–min standardized, respectively. This standardization process involved subtracting the minimum value of each dataset from its feature vectors and then dividing by the range between the maximum and minimum values.

### Model Architectures—Construction of the Domain Adversarial Neural Network

Domain adaptation is the ability to transfer models from the source domain to the target domain by learning the differences between the source and target domains. This study presents a model constructed using the domain adversarial neural network, originally proposed by Ganin et al. in 2016,^[^ ^36^ ^]^ which comprises a feature extractor, domain discriminator and predictor. Among them, the feature extractor transforms the input data into abstract and meaningful high‐level features, facilitating the mapping of the original feature space to a more discriminative and lower dimensional space. This transformation enhances the model's understanding of the data, making it easier for downstream classifiers to fit the features and labels effectively. The domain discriminator distinguishes the source and target domains by analyzing the extracted features. It is trained against the feature extractor to ensure that it extracts invariant representations, realizing the extracted features consistent with the distributions in both the target and source domains. The predictor categorizes the samples based on their invariant representations to ensure that they contain useful information for the classification task. Based on this structure, the optimization of our model was to achieve accurate classification by extracting invariant representations. This approach mitigates the issues caused by covariate shifts, where differences between the two domains may make a model trained on source domain data unsuitable for the target domain. The loss function of the model is as follows:

(3)

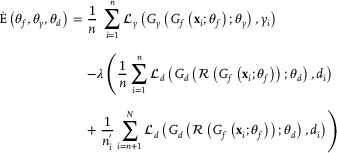

where *n* is the sample size of source domain, 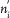 is the sample size of target domain, N is the sample size of both the two domains. *L_y_* is the loss function of the classifier, *G_y_* (*x*; θ_*y*_) is the structure and parameters of the classifier, *G_f_* (*x*;θ_*f*_) is the structure and parameters of the feature extractor, λ is the hyperparameter that determines the scale of the model to the loss of the domain discriminator, *L_d_* is the loss function of the domain discriminator, *G_d_* (*x*; θ_*d*_) is the structure and parameters of the domain discriminator, and *R*( ) is the gradient reversal function, through which the end‐to‐end learning of the model can be realized.

In our method, the model takes simulated data characteristics from the source domain and real data characteristics from the target domain as inputs and outputs classification and discrimination results that identify sample categories in both domains. Since the feature extractor plays a crucial role in determining the model performance, this study aimed to design and compare its structure for a more efficient and effective capture of features relevant to selective sweep detection and classification. We applied a filter with a convolution kernel size of (1,5) and a pooling layer with a pooling size of (1,4) after the input layer in our model to enhance its focus on the sequence series information within each row of the input feature matrix. Subsequently, we utilized a filter with a convolution kernel size of (3,1) and a pooling layer with a pooling size of (2,1). We then used two bottleneck modules to capture the features between the channels and reduce the number of parameters in the model. Accordingly, we used a 2D convolutional neural network (2D CNN) architecture to build the feature extractor.

The classifier is composed of four fully connected layers, with a batch normalization layer (BN layer) added after two of the fully connected layers to improve the model's generalizability. The first three fully connected layers adopt ReLU as the activation function, while softmax is used in the last layer to convert the model output into a probability distribution. Simultaneously, dropout is implemented in the fully connected layer to prevent model overfitting. The discriminator's architecture parallels the classifier (Figure 2). Hyperparameter (𝜆) and discriminator's structure determine the ability of the model to extract invariant representations. However, an excessive focus on extracting invariant representations could disregard the significance of features in classification. Therefore, on the condition that the accuracy of the model on the test set did not decrease significantly, λ was fixed at 1, and discriminators composed of 1, 2, 3, and 4 fully connected layers were tried. Finally, a domain discriminator composed of 3 fully connected layers was adopted based on the performance of the model on the test set. To date, the construction of a model based on a domain adversarial neural network has been accomplished (Figure S19, Supporting Information).

### Model Architectures—The Deep Ensemble Strategy

To enhance the stability of DASDC, we further employ a deep ensemble strategy. The specific procedure is as follows: (i) M DASDC models are randomly initialized and trained respectively; (ii) the trained models are used for prediction, and the mean of their predictions from M models is taken as the final result. Its formula can be expressed as:

(4) $$p\left(y|x\right)\;=M^{-1}\;\sum_{m\;=\;1}^{M}p_{\theta_{m}}\left(y|x,\theta_{m}\right)$$

where *p*(*y*∣**x**) is the output distribution of the deep ensemble strategy, M is the number of ensemble models, and $p_{\theta_{m}}\left(y|x,\theta_{m}\right)$ is the output distribution of the *m_th_* model with an initialization parameter θ_*m*_.

### Data Simulation

To systematically evaluate the performance of the method, discoal software was employed to simulate experimental data, which was also integrated into DASDC software to facilitate its application across various species.^[^ ^67^ ^]^ Referring to the previous data simulation of selective sweeps, based on whether selection occurs, the selected types of favorable alleles and their positions in the genomic fragment, the classes of selective sweeps simulated in this study include: (i) a hard sweep (or soft sweep) that occurred within the genomic segment 0.475 L to 0.525 L (L is the length of chromosome segment), which was defined as a hard sweep (or soft sweep); (ii) a hard sweep (or soft sweep) that occurred outside the genome segment 0.475 L to 0.525 L, which was defined as a linked hard sweep (or linked soft sweep); (iii) neutrality, which was defined as the occurrence of no selection events within the genome segment.

Then, we simulated the selective sweep data of one source domain and 16 target domains to evaluate the performances of different methods in situations where there is a mismatch in population genetic parameters between the source and target domains. We also simulated an ideal scenario in which the parameters of both the target and source domains were uniform to assess the performance of DASDC and existing methods. The detailed simulated process of mismatched and ideal scenarios was further elaborated in Supporting Information. To access the robustness of DASDC, the data of background selection, recombination rate heterogeneity, low recombination rate, missing genomic regions, and label mismatch were simulated as described in Supporting Information. To investigate the performance of DASDC in real data, we chose the CEU population, LW pigs, and Anopheles BFS population and used discoal software to simulate three sets of corresponding selective sweep genome data for training model. Each dataset comprised 15000 cases, with 12000 cases were used as the training set (2400 cases per class), 1500 cases were used as the validation set (300 cases per class), and 1500 cases were used as the test set (300 cases per class). The detailed parameters were further elaborated in the section on the application of detecting and classifying selective sweeps to a real dataset.

### Comparison to Other Methods

Based on the simulation data and the strategy of transductive learning in domain adaptation, DASDC was systematically compared with 5 classical statistical methods, deep learning‐based partialS/HIC and machine learning‐based Trendsetter. Here, the classical statistical methods used for comparison, including iHS, nSL, Tajima's D, SweeD^[^ ^68^ ^]^ and ω, basically cover the typical characteristics of all selective sweeps. Compared with machine learning‐based methods, these methods have low computational complexity and are easy to use. The Trendsetter approach employs 6 summary statistics and utilizes a multiple regression method for model construction, effectively leveraging multidimensional information. Trendsetter is considered representative of machine learning‐based methods. PartialS/HIC uses 89 summary statistics, converts the selective sweep detection and classification problem into a computer vision problem, and uses an AlexNet‐style convolutional neural network framework for model training. Because partialS/HIC has good classification performance, we chose this method to represent deep learning‐based methods.

The receiver operating characteristic (ROC) curve and the area under the receiver operating characteristic curve (AUC) were used as evaluation metrics to assess the model performance. For the 5 traditional statistical methods, because they did not have classification performance, we regarded the linkage and neutral classes as negative classes and the selection classes as positive classes. The extreme values of the statistics between the genome fragment 0.45–0.55 were taken as the predicted values of the samples for evaluation.

### Assessment of DASDC

Based on the simulation data, the robustness of DASDC was further investigated in some common technical and evolutionary hurdles that could lead to erroneous detection of sweeps, including background selection, recombination rate heterogeneity, low recombination rate, missing genomic regions, and label mismatch. This process was detailed in Supporting Information for robustness assessment. Similarly, the model is trained using the strategy of transductive learning (Figure S20, Supporting Information).

To assess which features were important for DASDC, we employ the smoothGrad method to generate a saliency map for all 40 summary statistics in this analysis. It is helpful for understanding how the model works from an input standpoint and ensuring that the predictive model is not perceived as a black box. This process was detailed in Supporting Information.

### Application of Detecting and Classifying Selective Sweeps to a Real Dataset

In this study, humans, pigs, and Anopheles were selected to evaluate the performance of DASDC on real data. Human data were obtained from the Utah Population in Northern and Western Europe released in Phase III of the 1000 Genomes database (www.internationalgenome.org). The parameters used in human CEU population simulation data were derived from the population genetic parameters provided by Schrider and Stdpopsim and the Human Out of Africa Model deduced by Tennessen in 2012^[^ ^24^, ^69^, ^70^ ^]^ (Table S5, Supporting Information). Domestic animal data were obtained from an LW pig population with a sample size of 269, of which 184 samples were obtained from the whole‐genome resequencing data of the National Center for Biotechnology Information (NCBI), and the remaining 85 samples were obtained from the whole‐genome resequencing data determined by our laboratory. Follow the standard workflow, GATK4.1.9 be used to SNP calling,^[^ ^71^ ^]^ and imputing by AGIDB.^[^ ^72^ ^]^ The parameters used by the LW pig population were inferred using Relate software (Figure S21 and Table S6, Supporting Information).^[^ ^73^ ^]^ *Anopheles gambiae* data were obtained from BFS population and were released during the first phase of the Thousand Mosquito Genome Project (https://www.malariagen.net/). The parameters used by the Anopheles BFS Anopheles population were simulated by Xue in 2021.^[^ ^33^ ^]^ DASDC was trained using the strategy of transductive learning in domain adaptation (Figure S20, for details see Supporting Information for domain adaptation).

## Conflict of Interest

The authors declare no conflict of interest.

## Author Contributions

Y.L.M. conceived the study. Y.L.M and S.H.Z. designed the experiments and provided the data. H.S., J.Y.C., and W.J.L. performed the experiments and analyzed the data. H.S., J.L.H., L.Z.F., S.H.Z., and Y.L.M. wrote and modified the paper. H.S. contributed in software development: HS. All authors reviewed and approved the final manuscript.

## Supporting information

Supporting Information

Supporting Information

## Data Availability

The source code of DASDC is available at https://github.com/soo‐h/DASweepDetect.
