# Supplementary material for: A Novel Approach Utilizing Domain Adversarial Neural Networks for the Detection and Classification of Selective Sweeps
Source: Adv Sci (Weinh). 2024 Feb 2;11(14):2304842. doi: 10.1002/advs.202304842 (PMC11005742; doi:10.1002/advs.202304842)
Supplement: Supplementary file 1 — Supporting Information [file ADVS-11-2304842-s002.pdf]

ADVANCED SCIENCE
Open Access

Supporting Information

for *Adv. Sci.*, DOI 10.1002/advs.202304842

A Novel Approach Utilizing Domain Adversarial Neural Networks for the Detection and Classification of Selective Sweeps

Hui Song, Jinyu Chu, Wangjiao Li, Xinyun Li, Lingzhao Fang, Jianlin Han, Shuhong Zhao and Yunlong Ma**

Supplementary Information for

A novel approach utilizing domain adversarial neural networks for the detection and classification of selective sweeps

***Hui Song*[1]*, Jinyu Chu*[1]*, Wangjiao Li*[1]*, Xinyun Li*[1,2]*, Lingzhao Fang*[3]*, Jianlin Han*[1,4,5]*, Shuhong Zhao*[1,2,6,*]*, Yunlong Ma*[1,2,6,*]**

[1] Key Laboratory of Agricultural Animal Genetics, Breeding, and Reproduction of the Ministry of Education & Key Laboratory of Swine Genetics and Breeding of the Ministry of Agriculture, Huazhong Agricultural University, Wuhan 430070, China

[2] Hubei Hongshan Laboratory, Wuhan 430070, China

[3] Center for Quantitative Genetics and Genomics, Aarhus University, Aarhus, 8000, DK

[4] CAAS-ILRI Joint Laboratory on Livestock and Forage Genetic Resources, Institute of Animal Science, Chinese Academy of Agricultural Sciences (CAAS), Beijing 100193, China

[5] Livestock Genetics Program, International Livestock Research Institute (ILRI), Nairobi 00100, Kenya

[6] Lingnan Modern Agricultural Science and Technology Guangdong Laboratory, Guangzhou 510642, China

*Correspondence: Yunlong.Ma@mail.hzau.edu.cn, shzhao@mail.hzau.edu.cn

This document includes:

Supplementary Methods

Reference

Supplementary Figures S1-S23

Supplementary Methods

1. The simulated data for performance assessment

1.1 The bias scenarios simulation

In this study, we simulated the selective sweep data of 17 scenarios. One scenario was used as the source domain for model training, while the remaining scenarios served as 16 target domains with different population genetic parameters from the source domain to evaluate the performance of DASDC and existing methods, respectively. The simulation parameters for the source domain were set as follows: a constant population history size, a sample size of 50 individuals, mutation rate (μ) uniformly distributed between 1.25e-08 and 2.5e-08, recombination rate (r) also uniformly distributed within the same range, selection coefficient (s) ranging uniformly from 0.01 to 0.1, and a genomic segment length set at 100 kb (**Supplementary Table S8**). The recombination rate (r) and selection coefficient (s) of these 16 target domains were chosen from a range of [3.125e-09, 6.25e-09, 5.9e-08, 1.0e-07] and [2.5e-04, 5.0e-04, 0.02, 0.04], respectively (**Supplementary Table S9**). Each target domain consisted of 1000 cases per category (200 cases per class). The source domain comprised 15,000 cases, with 12,000 cases were used as the training set (2400 cases per class), 1,500 cases were used as the validation set (300 cases per class), and 1,500 cases were used as the test set (300 cases per class).

1.2 The ideal scenarios simulation

We simulated an ideal scenario in which the parameters of both the target and source domains were uniform to evaluate the performance of DASDC and existing methods. The CEU demographic simulation dataset (**Supplementary Table S5**) was used as the source domain to train Trendsetter and partialS/HIC. The target domain was simulated using the same parameters and demographics as the source domain, and all methods were evaluated in the target domain. The target domain consisted of 1000 cases per category (200 cases per class). The source domain comprised 15,000 cases, with 12,000 cases were used as the training set (2400 cases per class), 1,500 cases were used as the

validation set (300 cases per class), and 1,500 cases were used as the test set (300 cases per class).

2. Detecting covariance shift between real and simulated data

In this study, we first conducted a validation study to examine the possible covariance shift between the simulated selective sweep data and the real data. Here, the simulation dataset consisted of CEU simulation data that included 2,400 cases for each of the following classes: hard sweeps, linked hard sweeps, soft sweeps, linked soft sweeps and neutral scenarios. In total, there are 12,000 cases of simulation data. The real data used the CEU population, and the genome was divided into 525,759 genomic fragments with 1 Mb windows and a step length of 50 kb. To keep the sample number equal to simulation data, we randomly selected 12,000 samples from the real data. Subsequently, both datasets underwent a transformation in which individual samples were converted into feature matrices through the process of feature engineering. The Kolmogorov-Smirnov test, PCA, and SVM were then used to detect covariance shift between the simulation and real data.

2.1 Two-sample Kolmogorov-Smirnov test

In this analysis, the Kolmogorov-Smirnov test was employed to evaluate the disparities between the two datasets across each feature. To determine whether the distribution difference can be eliminated through distribution transformation, we applied Cox-Box standardization to both simulated and real genomic data features using scipy.stats.boxcox from the Python scipy library, resulting in transformed features that closely follow a normal distribution. After the transformation, we further normalized the data using Z-score standardization to ensure that each feature approximately follows a standard normal distribution. Finally, a two-sample Kolmogorov-Smirnov test was conducted on these 40 paired feature vectors to determine the statistical significance of their differences. Considering the sensitivity of the Kolmogorov-Smirnov test at high sample sizes, Mann-Whitney U test was also used to evaluate the disparities between the two datasets across each feature.

2.2 Detecting covariance shift using PCA and SVM

The feature matrix, based on 40 summary statistics, was used in this analysis following the strategy outlined in section 5.1.2 for genomic selective sweep feature engineering construction. We flattened each feature matrix into a feature vector for PCA and SVM because both methods require one-dimensional input vectors. For PCA, we conducted the analysis directly on 24,000 samples, which included 12,000 from simulated data and 12,000 from real data. The dataset for SVM was constructed by sampling 12,000 cases from both the real and simulated data. Then, the real genomic vectors were labeled as positive cases, while the simulated data vectors were labeled as negative cases in this analysis. The combined dataset was shuffled and then divided into a training set and a test set in an 8:2 ratio. Then, an SVM model was trained to detect the covariance shift between the simulated selective sweep data and the real data. To eliminate the influence of SVM model-intrinsic biases on the results, we conducted two control experiments following the same principles. The first control experiment randomly labeled 12,000 real genomic samples as positive or negative instances in a 1:1 ratio and then divided them into training and testing sets in an 8:2 ratio. The second control experiment used 12,000 simulated samples as the dataset, employing the same processing strategy. We then trained the two SVM models using the training dataset and evaluated them on the test dataset. The SVM training process was completed using sklearn.svm.SVC with a radial basis function (RBF) kernel.

3. Model architectures

3.1 The calculation of summary statistics

As described in the method titled "The choice of statistics for extracting the selective sweep feature", we utilized 40 summary statistics to capture the features indicating selective sweeps (**Supplementary Table S1**). Our new model was trained using a simulated dataset of 1Mb. Therefore, when applied to real genome data, the genome was also divided into 1Mb genomic fragments with a step length of 50kb. Accordingly, the computation of statistics was based on a genomic fragment with a length of 1Mb,

and each statistic was calculated using the SNP windows division strategy described in section "5.1.2 Genomic selective sweep feature engineering construction". Among them, ϕ, κ, SAFE and HAF were calculated in each window and their 1st to 4th statistical moments and the maximum value were used as the eigenvalues of the selective sweep for the construction of the feature matrix. Similarly, the 1st to 5th statistical moments and the entropy of the site frequency spectrum were also used as the eigenvalues of the selective sweep for the construction of the feature matrix. Note that iHS, nSL, and ΔiHH were calculated at the whole genome range in real data to reduce calculation complexity and effectively utilize long-range extended haplotype homozygous information.

3.2 The choice of default parameters in DASDC

The parameters k and n determine the way features are described, i.e., the number of SNP windows and the number of SNPs per window used for summary statistics calculation. Therefore, we experimented with various combinations of k from the set [10, 25, 50] and n from the set [10, 50, 100, 200], resulting in a total of 12 scenarios. Nucleotide diversity was used as our statistical metric. At k=25 and n=200, we observed that the selective sweeps were well captured (**Supplementary Figure S22**). Therefore, we chose these parameters as our default settings. As for parameter m, since it can be considered as a correction for the positional information of n, we used the setting m=n, i.e., m=200.

4. Verification of the feature extractor in DASDC

4.1 Comparison of the classification accuracy of the different feature extractors

To evaluate the efficacy of DASDC's feature extractor in enhancing data information utilization within the model, we compared it with other classical structures such as 2-layer LSTM RNNs, 1D CNNs and 2D CNNs used as feature extractors. To control for extraneous variables, we held constant the discriminator and domain classifier components of the model while only modifying the feature extractor. Here, the dataset utilized for model training and performance evaluation comprised both simulated and

real CEU data. The model was trained within 50 iterations, using a learning rate that linearly decayed from an initial value of 0.01. Stochastic Gradient Descent (SGD) was used as the optimizer. To avoid overfitting, we monitored the loss on the validation set and terminated the training prematurely if there was no discernible reduction in loss for 5 consecutive iterations. Since the random initialization of a model can introduce variability into its final performance, we conducted 10 training runs for each model to mitigate the impact of this randomness on the evaluation results. During each run, we selected the iteration parameter with the lowest loss on the verification set as our final model and averaged the performance of these 10 models generated across all runs to represent overall model performance. The structure of the feature extractor for comparison was shown in **Supplementary Figure S23**.

4.2 Ablation experimental

To assess the impact of model components on performance, we conducted ablation experiments to demonstrate that our initial two layers of structures can enhance performance for the detection of selective sweeps. Here, we have developed two models for comparative testing based on the current architecture. The first model removes the initial two convolutional layers, while in the second model, we maintain the number of filters in these layers but change their kernel size to 3x3 and adjust the pooling layer size to 2x2 to evaluate its impact on overall performance.

4.3 Confidence computation

In this analysis, we utilized confidence as a measure to assess the level of certainty in the model's predictions. Specifically, for classification tasks, it quantifies the average probability that the model assigns to the correctly predicted class. To calculate the overall confidence metric for the dataset, we computed the average of these individual probabilities assigned to the correctly predicted classes. Formally, N is the number of correctly classified samples and p_i is the maximum value of the softmax layer output for the i th correctly classified sample, the overall confidence is:

$$\text{Confidence } = \frac{1}{N}\sum_{i=1}^{N} p_i$$

4.4 significance tests

We conducted a one-tailed t-test evaluation of our model and the 2D Convolutional Neural Network (CNN). A significance analysis of loss and accuracy metrics was carried out based on ten repeated training for each model. Additionally, we conducted a Shapiro-Wilk test to assess the normality of the four sets of data, all of which returned p-values greater than 0.05, affirming the validity of employing t-tests.

5. The application of domain adaptation in DASDC

Domain Adaptation is the ability to transfer models from the source domain to the target domain by learning the differences between the source domain and the target domain, which falls under the category of transfer learning[1]. In model application, there are two major classifications depending on the target samples: the first is known as the transductive setting, which predicts labels for specific target samples; and the second is the inductive setting, used to predict labels for new samples from the target domain (**Supplementary Figure S20**). The major distinction between the settings lies in the dataset used for evaluation: the transductive setting focuses on a specific set of target samples (**Supplementary Figure S20**, blue dotted arrow), while the inductive setting evaluates the model's ability to process new, previously unknown samples (**Supplementary Figure S20a,** red dotted arrow). Empirically, the inductive approach is impractical for analyzing real populations due to the unknown evolutionary processes of organisms. Therefore, for the detection and classification of selective sweeps in real populations, we have adopted the transductive setting in this study.

The training process of the model for real genome data was illustrated in **Supplementary Figure S20b**. Referring to previous studies[2], we obtained population genetic parameters of a real observed population through parameter inference and literature mining, and simulated labeled pseudo-data as the source domain. Subsequently, we divided the simulated data into training, validation, and test sets in an

8:1:1 ratio. The target domain was constituted by sampling real genomic data, adhering to a random extraction criterion that matched the sample size of the training set from the simulated data (n=12000). According to the Law of Large Numbers, the distribution of this target domain should align with that of the real genome, making it essentially a transductive setting despite not using the whole real genome as the source domain. We then trained the DANN model, evaluating its performance on the test set.

Five models were trained, and abnormal models were identified and excluded based on significant performance deviations on the test set by **Z-score test (threshold set at 2).** In the absence of anomalous models, these five were integrated (refer to “The deep ensemble strategy”); otherwise, the training process was repeated until five models were obtained and subjected to the same procedure. Finally, the ensemble model was applied for the detection and classification of selection signals in real genomic data.

For both the source and target domains, which consisted of simulated data (performance evaluation based on simulated data method), the training process was illustrated in **Supplementary Figure S20c**. The data for both domains were generated using corresponding population genetics parameters. The subsequent training process and prediction outcomes were consistent with training process of the model used real genome data.

6. Further comparison for machine learning methods

To control the performance difference resulting from the use of summary statistics, we constructed each method by employing DASDC's summary statistics and feature engineering. Then, the performance of DASDC, partialS/HIC and Trendsetter were compared under two scenarios: constant demographic and CEU demographic. For the constant demographic scenario, the simulation parameters were as follows: mutation rate (μ) ~ U (1.25e-08, 2.5e-08), recombination rate (r) ~ U (1.25e-08, 2.5e-08), selection coefficient (s) ~ U (0.01-0.1), and a genome segment length set at 100 kb (**Supplementary Table S8**). Using these parameters, we generated 3,000 cases of simulated hard sweeps, hard linkage sweeps, soft sweeps, soft linkage sweeps, and

neutral each. The dataset was divided into training, validation, and test sets in the ratio of 8:1:1, respectively. The datasets were then processed using the feature engineering protocol of DASDC, with the processed training and validation sets used to train the three methods, followed by evaluation on the processed test set. To mitigate the influence of stochastic factors, each method was independently trained 10 times, with the optimal model being selected for performance evaluation.

For the CEU demographic, we utilized simulated data for the CEU population (parameters can be found in **Supplementary Table S5**), following the same procedure as with the constant demographic to evaluate between methods.

7. Robustness assessment

To further evaluate the robustness of DASDC, we investigated 5 potentially important factors, including background selection, heterogeneity in recombination rate, low recombination rate, missing genomic regions, and label mismatch. In this analysis, the model training followed the flow shown in **Supplementary Figure S20c.**

7.1 The robustness for background selection

To explore the robustness of DASDC in background selection (BGS), we simulated 4 scenarios of background selection following the strategy proposed by Mughal and DeGiorgio [3]. Here, we used the SLiM3 software to simulate 500 cases for each scenario[4].

The genomic arrangement for the functional elements in the scenarios (i) and (ii) follows the order of 5'UTR, with 10 exons and 9 introns arranged alternately, and ends with 3'UTR, as detailed in Cheng et al. [5]. The length of each intron, exon, 5'UTR, and 3'UTR was defined as 1,000, 100, 200, and 800 nucleotides, respectively, based on their average sizes in the human genome as reported by Mignone et al. and Sakharkar et al. [6, 7]. We assigned 75%, 50%, and 10% of mutations occurring in exons, UTRs, and introns as deleterious, respectively. The selection coefficient for deleterious mutations was fixed at s=-0.1. The mutation rate was set to 1.875e-8. The recombination rates for these two scenarios were set at 1.875e-8 and 1.875e-9, which

correspond to normal and low recombination rates under BGS, respectively. For the scenarios (iii) and (iv), we randomly extracted 1.1Mb regions from the human genome and identified sites within those regions that were either listed in the phastCons database[8] or found within an exon in the GENCODE database [9]. These detected sites were then defined as the loci of purifying selection in the simulated genomic fragment. It is assumed that mutations at these sites are 25% neutral and 75% under selection, with the selection coefficients drawn from a gamma distribution with a mean of -0.0294 and a shape parameter of 0.184[10]. Mutation rates were sampled uniformly from the range (1.25e-8, 2.5e-8). The recombination rates for these scenarios were set to be uniformly drawn from (1.25e-8, 2.5e-8) and (1.25e-9, 2.5e-9), respectively, corresponding to normal and low recombination rates under empirical BGS.

Based on the simulation data of the above 4 scenarios, we evaluated the robustness of DASDC for background selection. The source domain data used in this analysis is the same as the source domain data from the 17 scenarios simulated data in the Method Section (5.3 simulations). The target domain data was simulated using the same parameters as the source domain, which included 4 non-neutral classes (hard, linked hard, soft, linked soft sweeps) consisting of 2400 cases each and a neutral class consisting of 1900 cases. Then, we integrated the 4 scenarios of BGS data into the neutral class to form the 4 target domain datasets for robustness evaluation.

Here, models were trained using the source and target domain in each scenario, and predictions were subsequently made for the BGS data of corresponding scenario to evaluate the model's robustness against BGS.

7.2 The robustness in missing genomic regions

To explore the robustness of DASDC in missing genomic regions, we processed simulation genomes with a 30% missing SNPs by masking 10 separate 3% SNP segments, following the strategy of Mughal and DeGiorgio[3]. Here, we evaluated the robustness of DASDC using two distinct scenarios: (i) without prior consideration of missing genomic data; (ii) with prior consideration of missing genomic data.

For scenario (i), the source domain employed the source domain from the 17 scenarios simulated data in the **Experimental Section** (5.2 Data simulation), while the target domain used the same parameters to simulate 2400 cases for each category. Then, we randomly masked 20% of the target domain data (480 cases) for each category, following the masking rule described above. For scenario (ii), both the source and target domain data are the same as scenario (i), but 20% of the data from each category in the source domain is masked (480 cases for each category).

Here, models were trained using the source and target domains in each scenario, and predictions were subsequently made for the missing data of corresponding scenario to evaluate the model's robustness against missing genomic data.

7.3 The robustness for recombination rate heterogeneity

To explore the robustness of DASDC for recombination rate heterogeneity, we simulated 3 distinct scenarios: (i) the target domain was comprised entirely of recombination rate heterogeneity data; (ii) the source and target domains were comprised entirely of recombination rate heterogeneity data; (iii) both domains included 50% recombination rate heterogeneity data. The recombination rate heterogeneity data was simulated using SLiM3, following the strategy of Johri et al. [11], where the genome was divided into segments with recombination rates sampled from a Gaussian distribution, and any negative values are truncated to zero. In our study, we divided the genome into three segments: 0-0.4L, 0.4L-0.6L, and 0.6L-1.0L, and set the mean of the Gaussian distribution for recombination rates at 1.85e-8 with a coefficient of variation of 0.5. All the other parameters were consistent with those of the source domain from the 17 scenarios outlined in the **Experimental Section** (5.2 Data simulation). Here, we simulated two batches of 12,000 cases of heterogeneous recombination data (2,400 cases per category), named heterogeneity dataset 1 and heterogeneity dataset 2. Additionally, we simulated 12,000 cases (2,400 per category) using the same parameters as the source domain and named it target dataset 1. Then, both source and target domains data for assessing the robustness of recombination rate heterogeneity was as follows:

Scenario	source domain	target domain
(i)	source domain dataset	heterogeneity dataset 2
(ii)	heterogeneity dataset 1	heterogeneity dataset 2
(iii)	50% source dataset 1 and 50% heterogeneity dataset 1	50% target dataset 1 and 50% heterogeneity dataset 2

Models were trained using the source and target domain in each scenario, and predictions were subsequently made for the heterogeneity data of corresponding scenario to evaluate the model's robustness against recombination heterogeneity. The assessment was conducted using ROC curves and confusion matrices.

7.4 The robustness for lower recombination rate

Our method was systematically evaluated across four scenarios with recombination rates reduced by 5-fold, 10-fold, 50-fold, and 100-fold relative to those in our source domain. The source domain employed the source domain from the 17 simulated scenarios in the **Experimental Section** (5.2 Data simulation). For the four scenarios in this analysis, each category was simulated with 2400 cases, with recombination rate parameters set at uniform distributions of U(2.5e-09, 5e-09), U(1.25e-09, 2.5e-09), U(2.5e-10, 5e-10), and U(1.25e-10, 2.5e-10), respectively. The other parameters were consistent with the source domain.

Given the DASDC's requirement for samples to have more than 250 SNPs, 83% of hard sweeps were below this threshold at a 50-fold reduction, and 91% with a 100-fold reduction. To maintain a balanced number of samples across categories, we adjusted the mutation rates upward by factors of 5 and 10 for the 50-fold and 100-fold scenarios, respectively.

Each model in the scenarios used the unlabeled data of that scenario as the target domain, and combined it with the source domain for training. Subsequently, the model made predictions on the respective target domain to assess the model's robustness to low recombination rates. The assessment was conducted using ROC curves and

confusion matrices.

7.5 The robustness for label mismatch

Real data has an a priori undetermined number of swept regions, which could be one major difference between the simulated data and the real data. We attempted to simulate this phenomenon through label mismatch, and evaluated the robustness of DASDC. Therefore, we simulated four distinct scenarios of between source domain and target domain: (i) Both features and labels are consistent (representing the ideal situation); (ii) Consistent features with mismatch labels (category imbalance); (iii) mismatch features with consistent labels (covariance shift); (iv) Both features and labels are mismatch (combining covariance shift and category imbalance).

For these four scenarios: the source domain data employed the source domain from the Experimental Section (5.2 Data simulation) from 17 different scenarios. For (i), the target domain data was simulated with parameters matching those of the source domain, with each category consisting of 2400 cases. For (ii), the five categories of target domain were simulated with proportions of 1:1:4:4:10 (hard : linked hard : soft : linked soft : neutral), using parameters identical to the source domain. For (iii), the target domain utilized simulations from the Experimental Section (5.2 Data simulation) with r=2.75e-08 and s=0.0075. For (iv), the target domain was simulated using the same ratios as in scenario (ii) and with the same parameters for the five categories as in (iii).

Each of the four models was trained using both the source domain and its corresponding target domain. Subsequently, they were used to make predictions on the respective target domain to assess the model's performance under each specific scenario. The performance assessment was conducted using ROC curves and confusion matrices.

8. Saliency maps

To understand which features of the input matrices are important for prediction, we analyzed the saliency maps under two different scenarios: biased and ideal data between the source and target domains.

In the ideal scenario, we used the source domain from the 17 scenarios in the Experimental Section (5.2 Data simulation) as the source domain. Using the same parameters (**Supplementary Table S8**), we further simulated 2400 cases for each of hard sweeps, linked hard sweeps, soft sweeps, linked soft sweeps, and neutral as the target domain. After training the model, we randomly selected 300 cases from each class in the target domain to calculate the average saliency maps for each category. The smoothGrad was used for calculation. In the biased scenario, the source domain data was the same as in the unbiased scenario, while the target domain datasets were generated based on the Experimental Section (**5.2 Data simulation**) with s=0.0075 and r=3.125e-9. The method for calculating the average saliency maps was consistent with the unbiased scenario.

The smoothGrad calculation process was completed using the saliency package, which is a third-party Python library. The significance of the SmoothGrad method lies in its ability to average the input gradients across multiple perturbations of the input data, which helps reduce noise and clarify the features that are considered most important by the model for making predictions[12]. This can provide more reliable and interpretable visual explanations of the model's behavior, particularly in high-dimensional data spaces.

Reference

[1]W. M. Kouw , M. Loog, *IEEE transactions on pattern analysis and machine intelligence* **2019,** 43, 766-785.

[2]X. Huang, A. Rymbekova, O. Dolgova, O. Lao , M. Kuhlwilm, *Nat Rev Genet* **2023,**

[3]M. R. Mughal , M. DeGiorgio, *Mol Biol Evol* **2019,** 36, 252-270.

[4]B. C. Haller , P. W. Messer, *Mol Biol Evol* **2019,** 36, 632-637.

[5]X. Cheng, C. Xu , M. DeGiorgio, *Mol Ecol* **2017,** 26, 6871-6891.

[6]F. Mignone, C. Gissi, S. Liuni , G. Pesole, *Genome biology* **2002,** 3, 1-10.

[7]M. K. Sakharkar, V. T. Chow , P. Kangueane, *In silico biology* **2004,** 4, 387-393.

[8]A. Siepel, G. Bejerano, J. S. Pedersen, A. S. Hinrichs, M. Hou, K. Rosenbloom, H. Clawson, J. Spieth, L. W. Hillier, S. Richards, G. M. Weinstock, R. K. Wilson, R. A. Gibbs, W. J. Kent, W. Miller , D. Haussler, *Genome Res* **2005,** 15, 1034-50.

[9]J. Harrow, A. Frankish, J. M. Gonzalez, E. Tapanari, M. Diekhans, F. Kokocinski, B. L. Aken, D. Barrell, A. Zadissa, S. Searle, I. Barnes, A. Bignell, V. Boychenko, T. Hunt, M. Kay, G. Mukherjee, J. Rajan, G. Despacio-Reyes, G. Saunders, C. Steward, R. Harte, M. Lin, C. Howald, A. Tanzer, T. Derrien, J. Chrast, N.

Walters, S. Balasubramanian, B. Pei, M. Tress, J. M. Rodriguez, I. Ezkurdia, J. van Baren, M. Brent, D. Haussler, M. Kellis, A. Valencia, A. Reymond, M. Gerstein, R. Guigo , T. J. Hubbard, *Genome Res* **2012,** 22, 1760-74.

[10]A. R. Boyko, S. H. Williamson, A. R. Indap, J. D. Degenhardt, R. D. Hernandez, K. E. Lohmueller, M. D. Adams, S. Schmidt, J. J. Sninsky, S. R. Sunyaev, T. J. White, R. Nielsen, A. G. Clark , C. D. Bustamante, *PLoS Genet* **2008,** 4, e1000083.

[11]P. Johri, C. F. Aquadro, M. Beaumont, B. Charlesworth, L. Excoffier, A. Eyre-Walker, P. D. Keightley, M. Lynch, G. McVean, B. A. Payseur, S. P. Pfeifer, W. Stephan , J. D. Jensen, *PLoS Biol* **2022,** 20, e3001669.

[12] A. Kapishnikov, T. Bolukbas, F. Viégas, M. Terry, *In Proceedings of the IEEE/CVF International Conference on Computer Vision,* 10.1109/ICCV.2019.00505.

Supplementary Figures

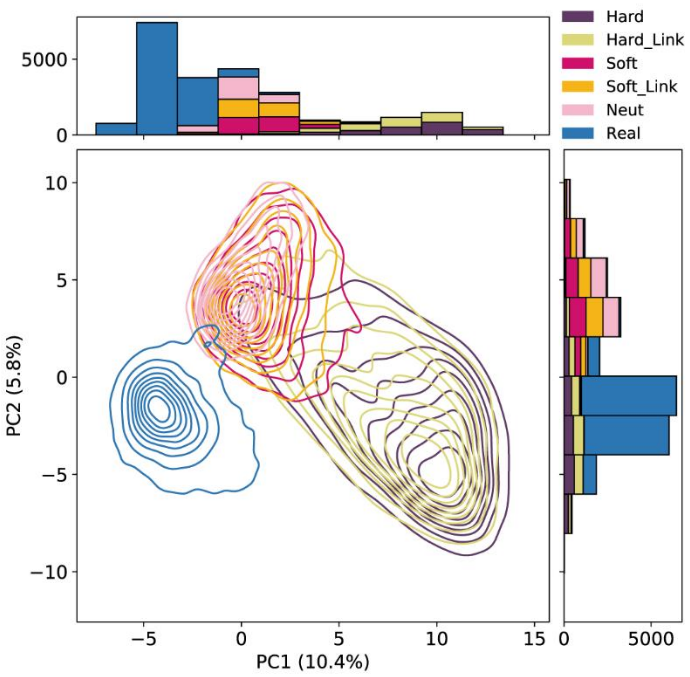

Figure S1 PCA results of simulated and real genome data. Real data is represented in blue and simulated data is split into five categories. The frequency histograms at the top and right of the graph represent the distribution of values of the sample along PC1 and PC2, respectively.

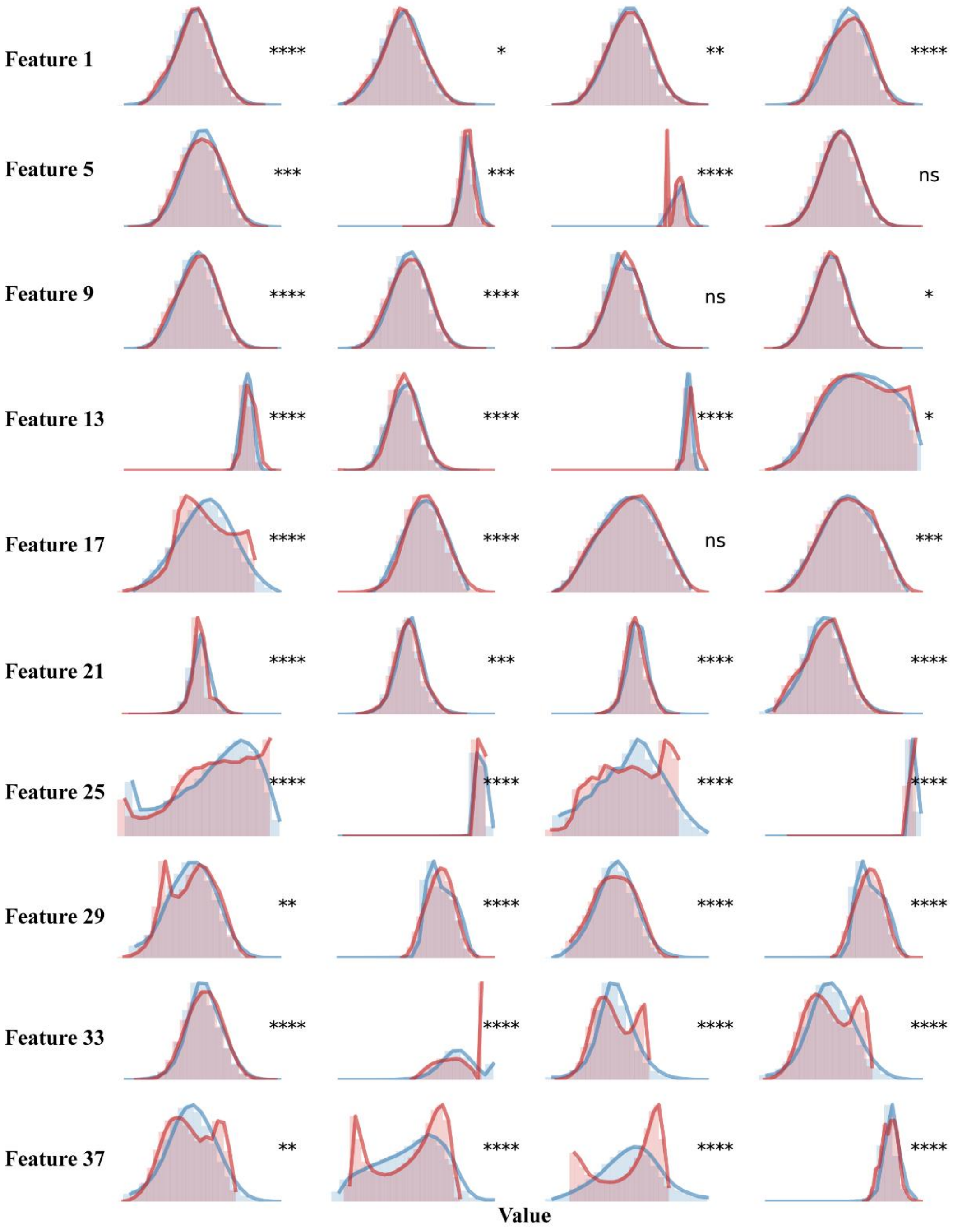

Figure S2 Probability distribution density between the 40 features of the real and simulated genomes of the CEU population. Red represents simulation data and blue represents real data. Significant test used Mann-Whitney U test. *, p ≤ 0.05; **, p ≤ 0.01; ***, p ≤ 0.05; ****, p ≤ 0.01.

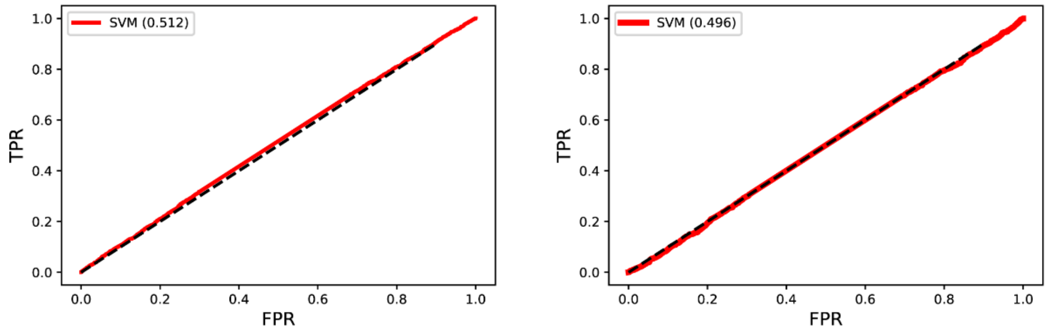

Figure S3 (a) The area under the receiver operating characteristic curve (AUROC) is approximately 0.496 for the discrimination results of SVM when distinguishing between two groups of real genome data. (b) The area under the receiver operating characteristic curve (AUROC) is approximately 0.512 for the discrimination results of SVM when distinguishing between two groups of simulated genome data.

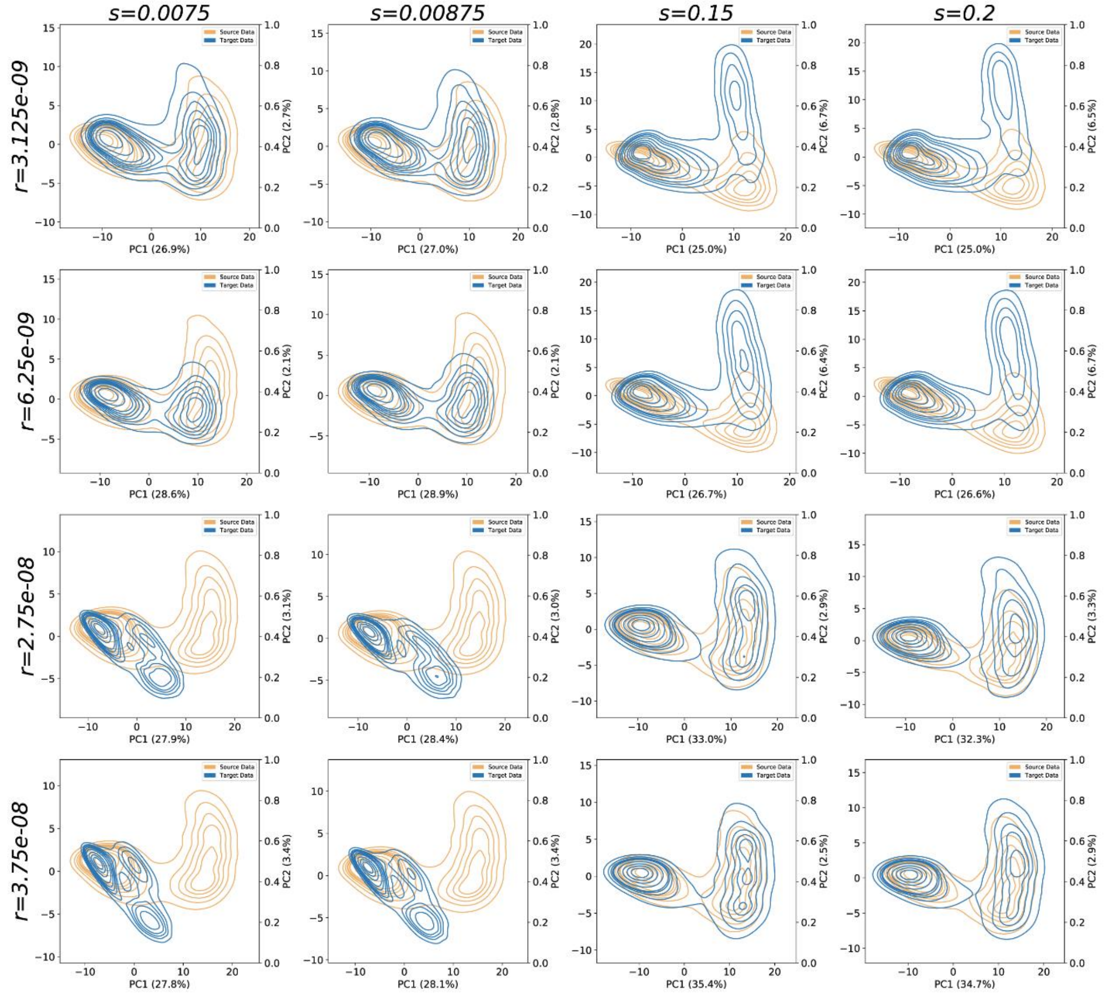

Figure S4 Assessment of the level of mismatch between the source domain and the target domain across 16 scenarios. The selection coefficient parameters and the recombination rate parameters are shown on the top and right of the figure, respectively. PC1 and PC2 are shown on the bottom and right borders, respectively.

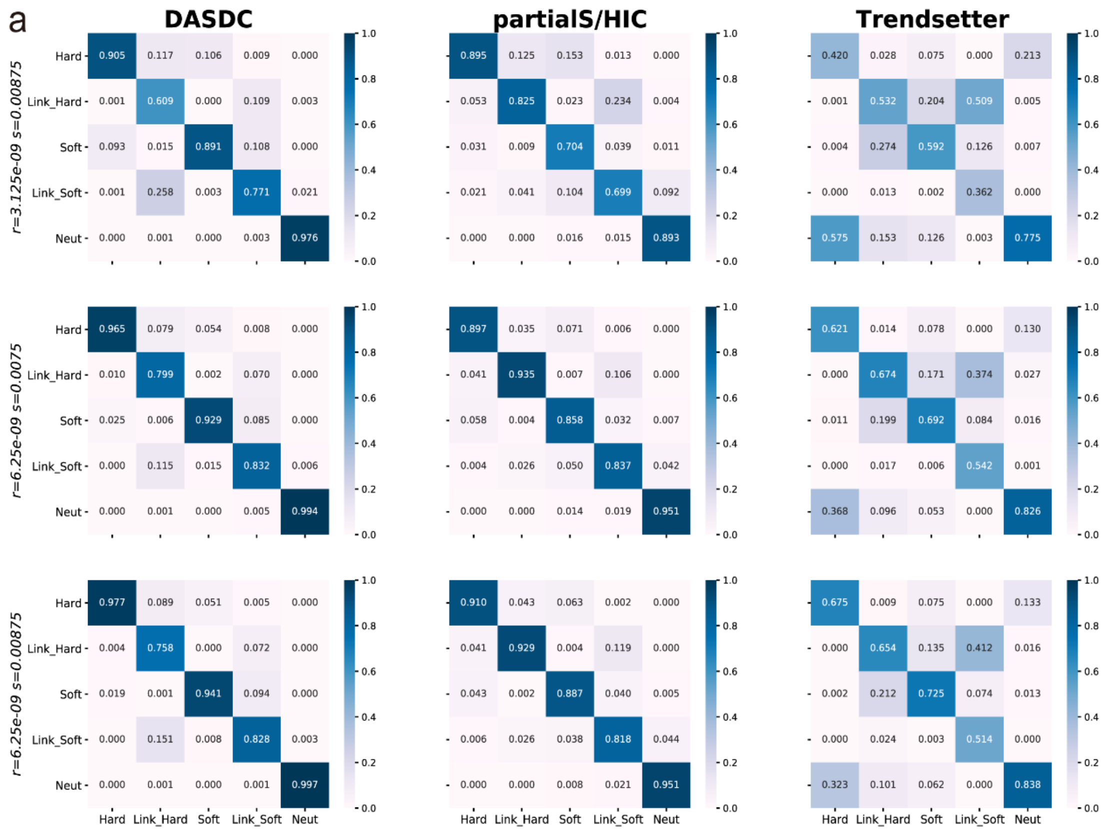

Figure S5 (Continued)

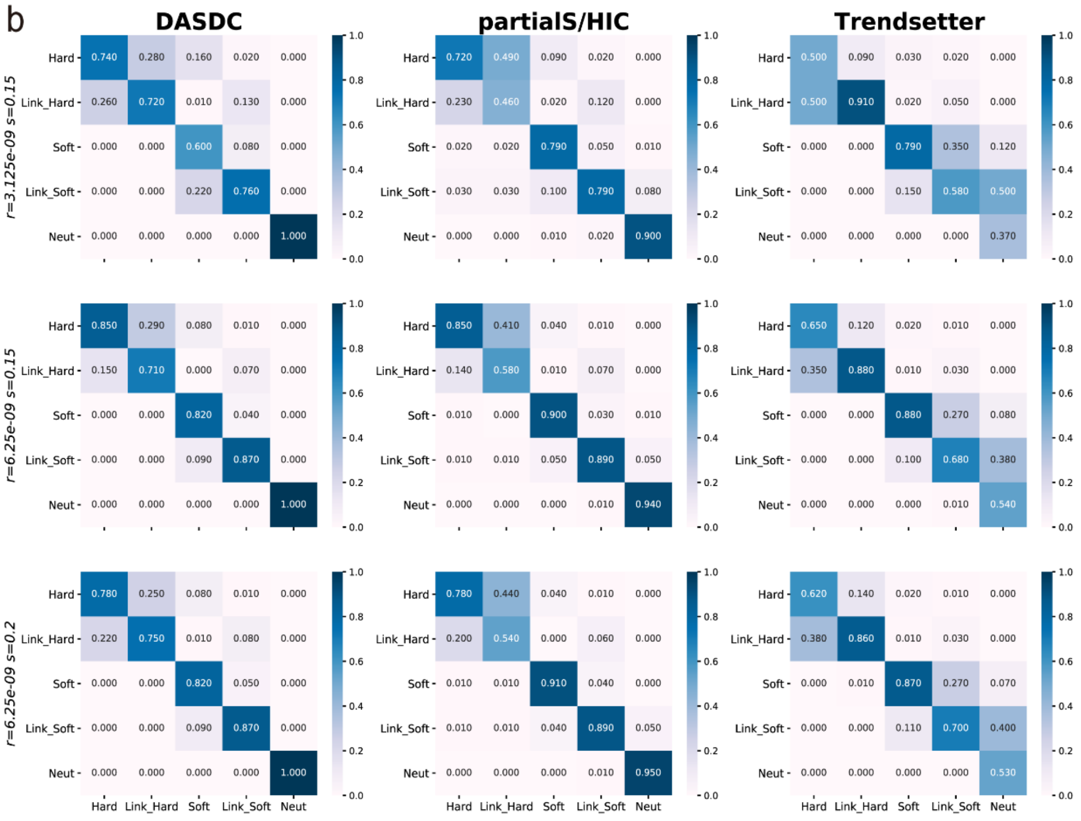

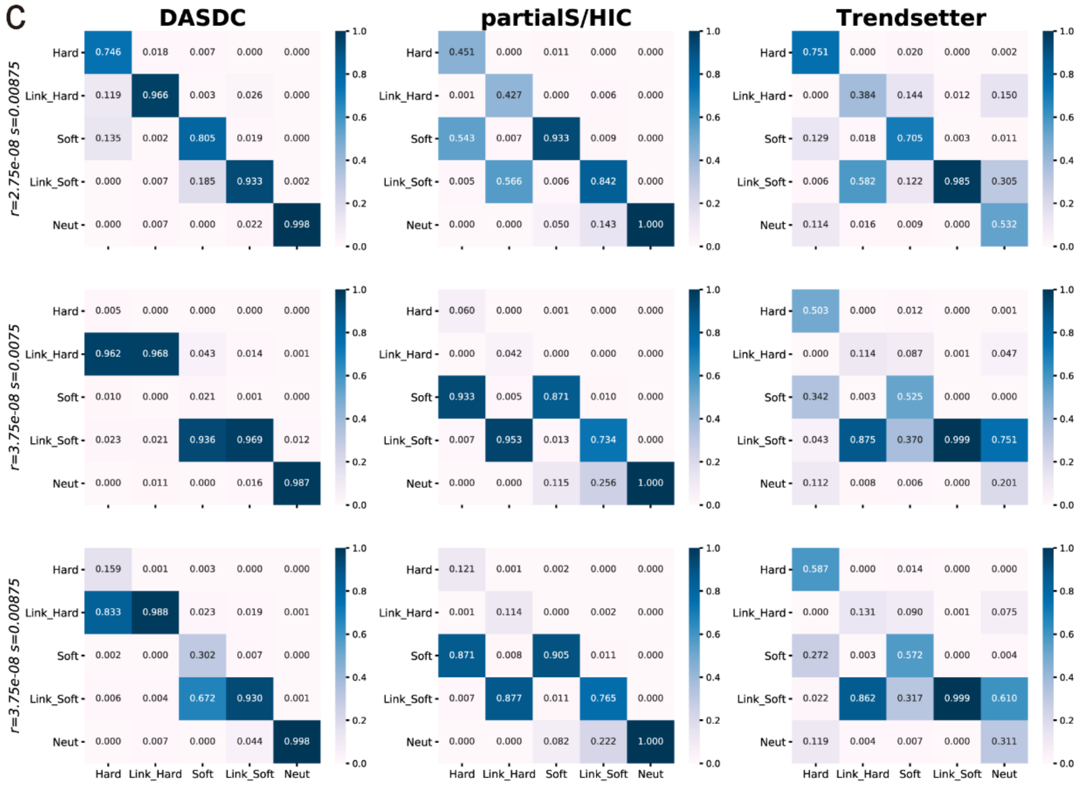

Figure S5 (Continued)

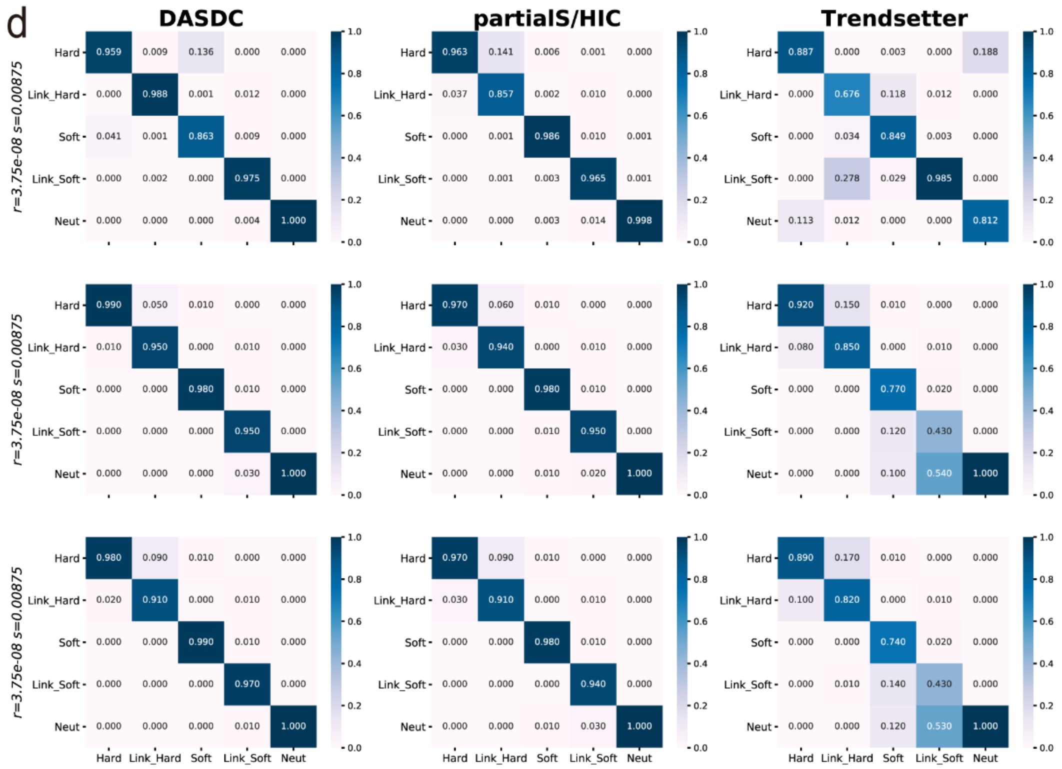

Figure S5 The confusion matrix of DASDC, partialS/HIC, and Trendsetter in four class situations. (a) Situation of lower recombination rate and lower selection coefficient

parameter than training situation. (b) Situation of lower recombination rate and higher selection coefficient parameter than training situation. (c) Situation of higher recombination rate and lower selection coefficient parameter than training situation. (d) Situation of higher recombination rate and higher selection coefficient parameter than training.

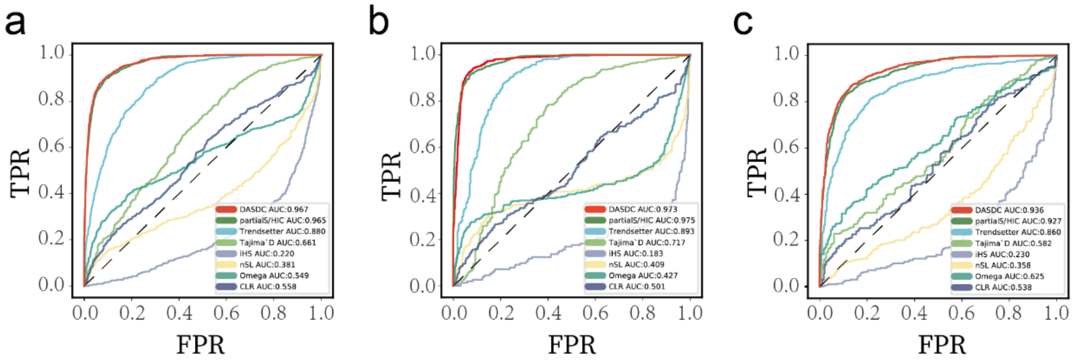

Figure S6 Performance comparison in ideal scenarios

ROC curve for 8 methods composed of different categories. (a) ROC curve when the dataset consists of hard sweep, soft sweep, linked hard sweep, linked soft sweep and neutral. (b) ROC curve when the dataset consists of hard sweep, linked hard sweep and neutral. (c) ROC curve when the dataset consists of soft sweep, linked soft sweep and neutral.

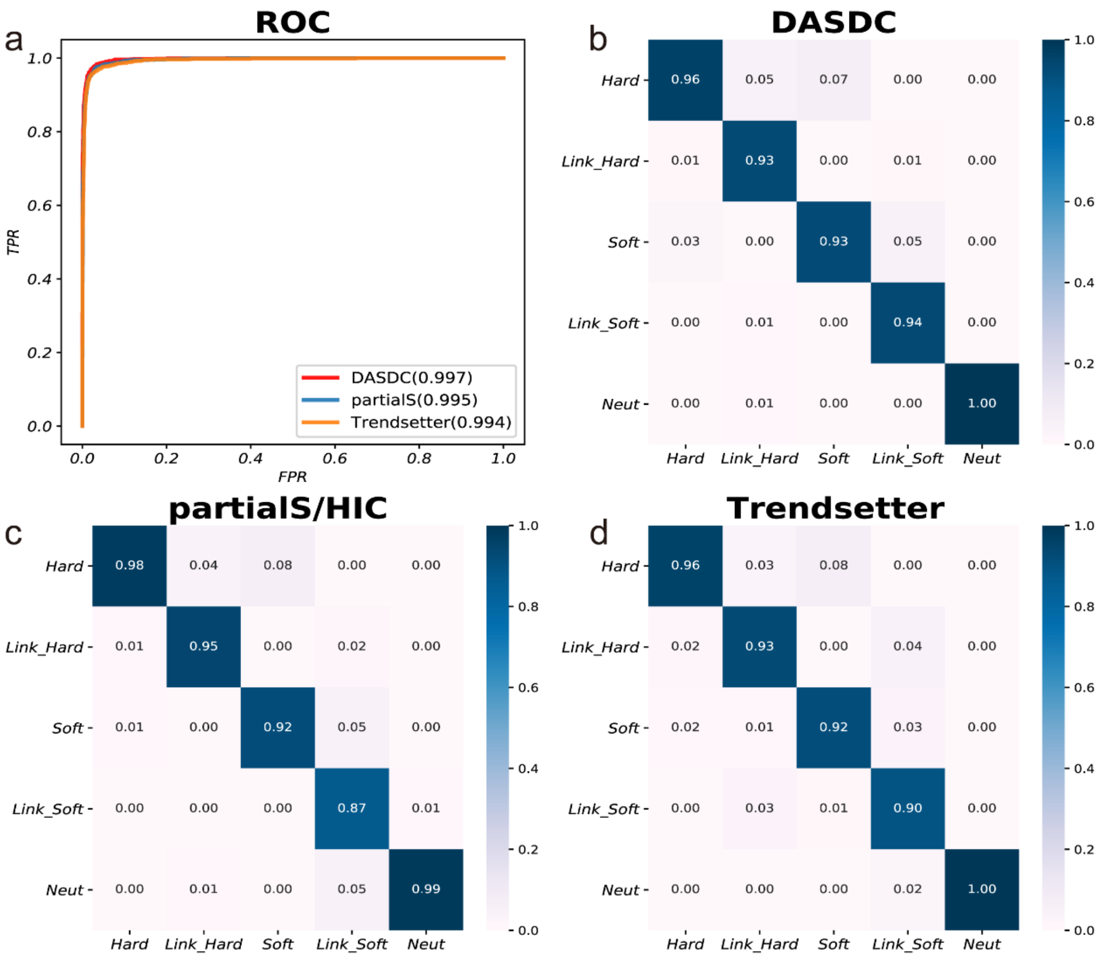

Figure S7 methods compare in constant demographic scenario DASDC's summary statistics and feature engineering. (a) ROC curve for 3 methods. (b) Performance of DASDC. (c, d) Performance of partialS/HIC and Trendsetter. Note that the horizontal coordinate is the true label and the vertical coordinate is the predicted label.

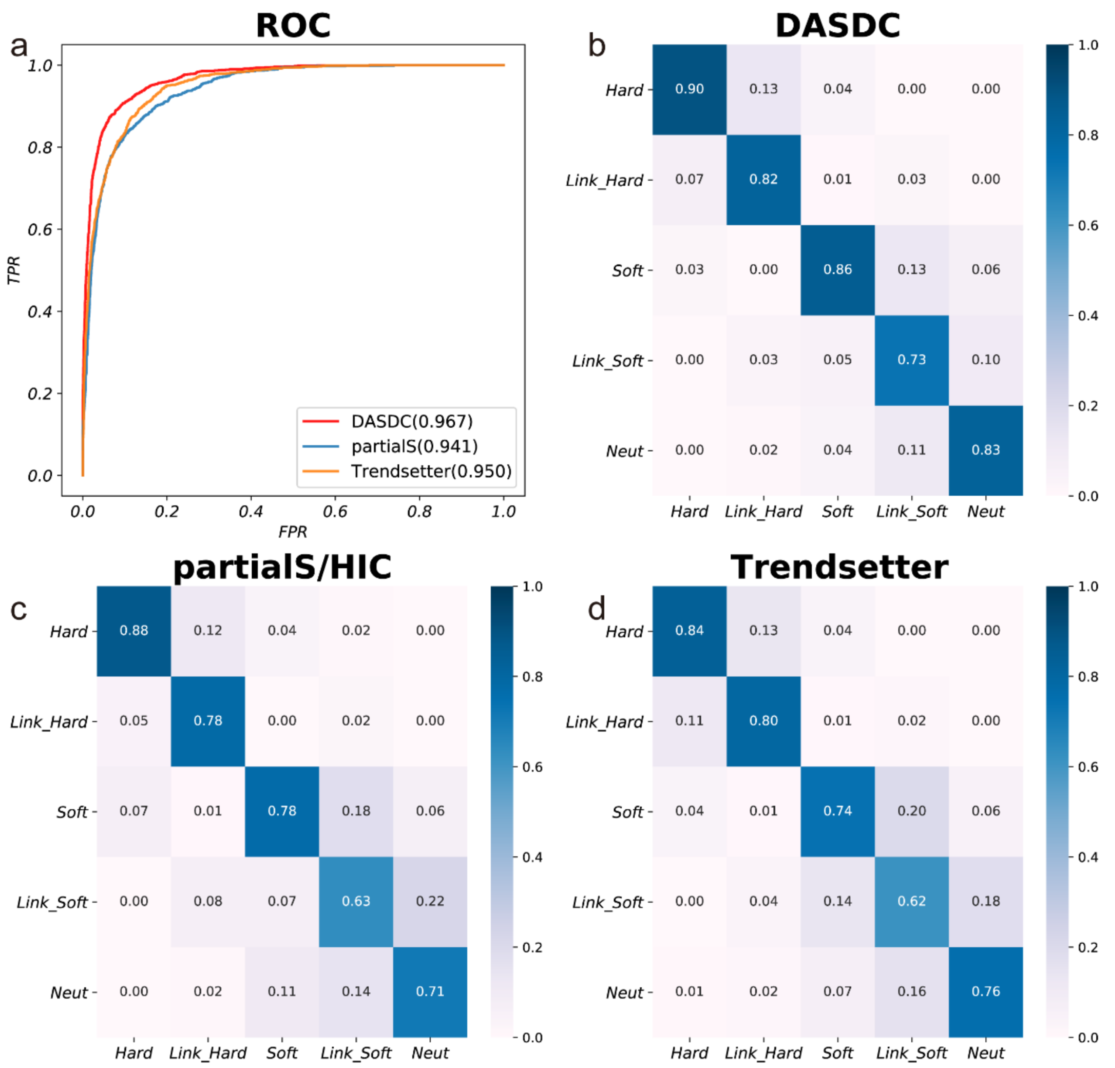

Figure S8 methods compare in CEU demographic scenario with DASDC's summary statistics and feature engineering. (a) ROC curve for 3 methods. (b) Performance of DASDC. (c, d) Performance of partialS/HIC and Trendsetter. Note that the horizontal coordinate is the true label and the vertical coordinate is the predicted label.

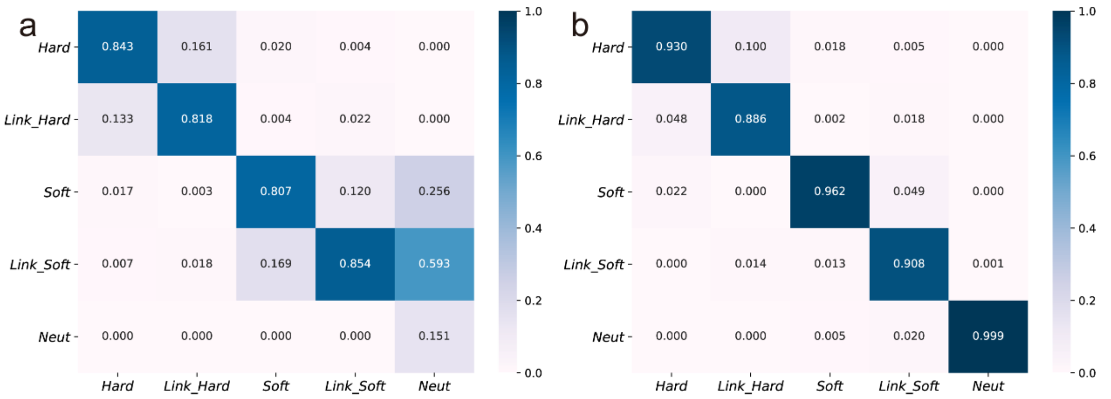

Figure S9 Confusion matrix of missing genomic data robustness. (a) Confusion matrix without considering missing data in the source domain. (b) Confusion matrix of considering 20% missing data in source target.

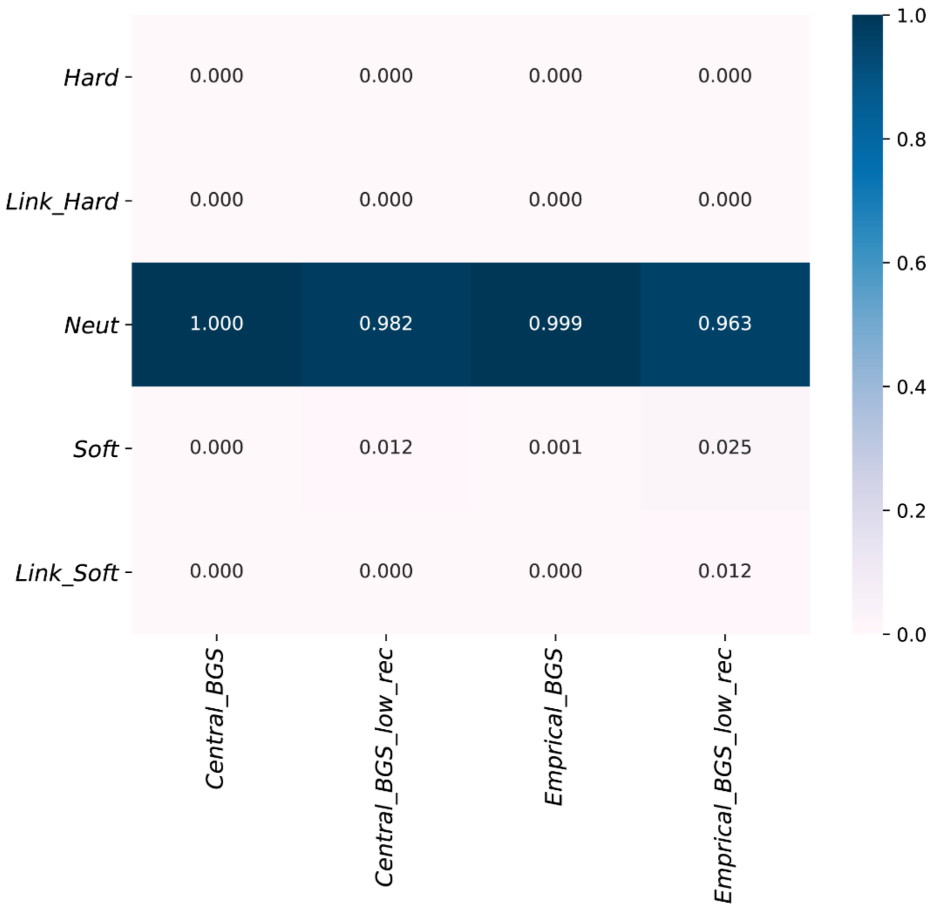

Figure S10 Confusion matrix of background selection robustness. Row labels indicate categories predicted by the model, while column labels represent scenarios of background selection.

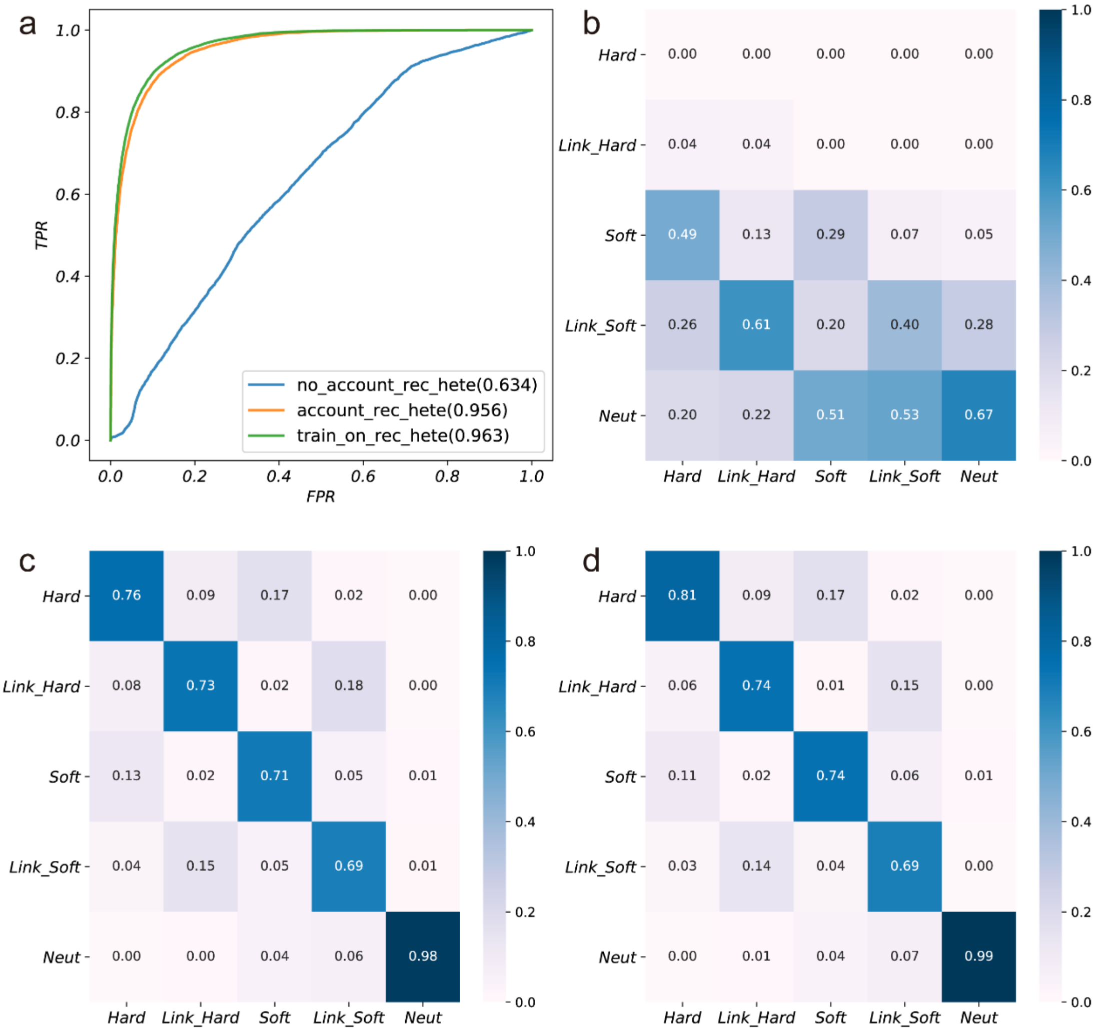

Figure S11 Assessment of robustness to recombination heterogeneity. The target domain consists entirely of recombination heterogeneity data. (a) ROC curves of DASDC under three scenarios: source domain without recombination heterogeneity data (blue line), source domain with 50% recombination heterogeneity data (orange line), and source domain entirely comprising recombination heterogeneity data (green line). (b) Confusion matrix when the source domain does not include recombination heterogeneity data. (c) Confusion matrix when 50% of the data in the source and target domains is recombination heterogeneity data. (d) Confusion matrix when the entire source domain comprises recombination heterogeneity data.

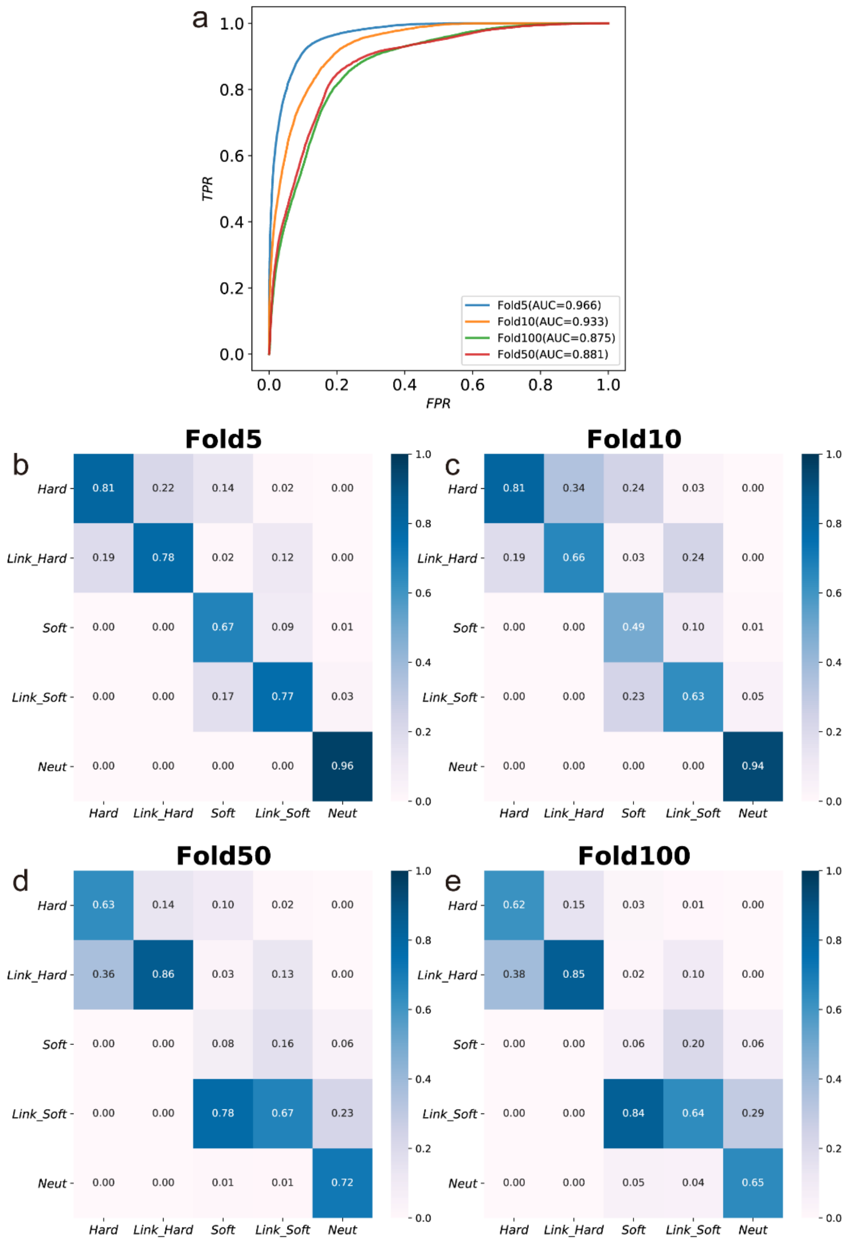

Figure S12 Assessment of robustness to low recombination rates. (a) ROC curves of DASDC under four recombination gradients. (b) Confusion matrix when the recombination rate in the target domain is 5 fold lower than in the source domain. (c) Confusion matrix when the recombination rate in the target domain is 10 fold lower than in the source domain. (d) Confusion matrix when the recombination rate in the target domain is 50 fold lower than in the source domain. (e) Confusion matrix when the recombination rate in the target domain is 100 fold lower than in the source domain.

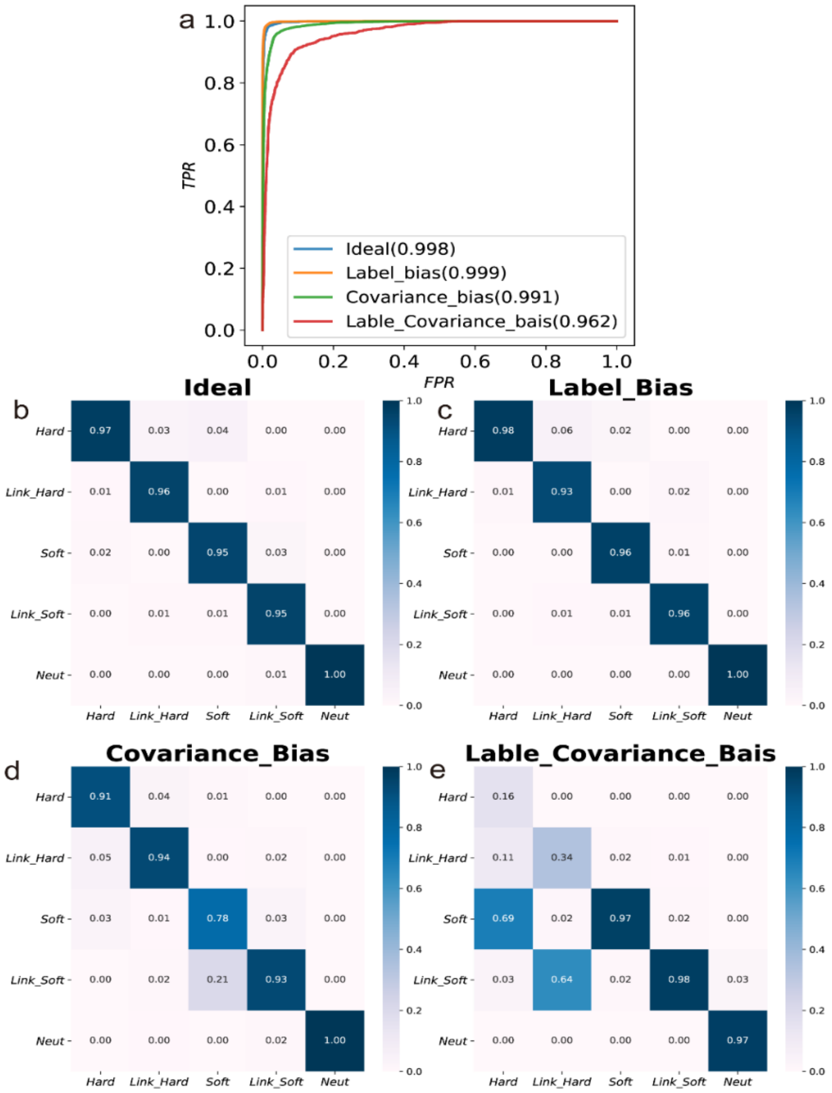

Figure S13 Robustness assessment of DASDC for label mismatch. (a) ROC curves of DASDC in ideal scenario (blue line), on label mismatch scenario (orange line), on covariance shift scenario (green line), and on both covariance shift and label mismatch scenario (red line). (b) Confusion matrix of DASDC in ideal scenario. (c) Confusion matrix of DASDC in label mismatch scenario. (d) Confusion matrix of DASDC on covariance shift scenario (e) Confusion matrix of DASDC on both covariance shift and label mismatch scenario.

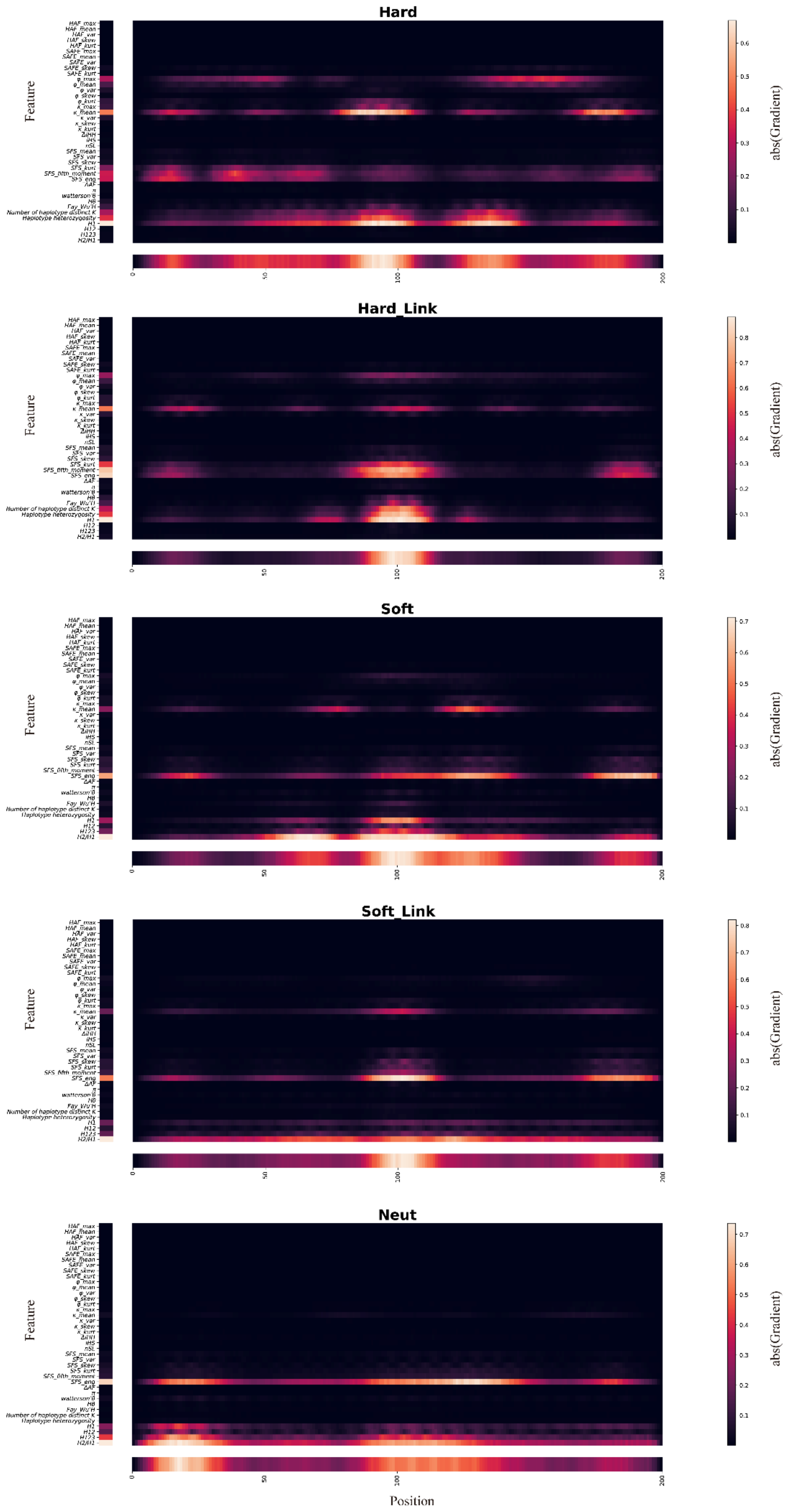
Hard
Hard_Link
Soft
Soft_Link
Neut
Feature
abs(Gradient)
Position
HAF_max
HAF_mean
HAF_var
HAF_skew
HAF_kurt
SAFE_max
SAFE_mean
SAFE_var
SAFE_skew
SAFE_kurt
SFS_mean
SFS_var
SFS_skew
SFS_kurt
SFS_fifth_moment
SFS_eng
watterson'θ
Fay_Wu'H
Number of haplotype distinct K
Haplotype heterozygosity
H1
H12
H123
H2/H1
0
50
100
200

Figure S14 Saliency maps in an ideal scenario. Each row represents a summary statistic, and each column represents a physical location interval. The brighter color represents importance of the corresponding statistic and physical location interval for the model's classification. The heatmap strip on the left is obtained by summing each row, indicating the importance of each summary statistic. The heatmap strip under each category is obtained by summing each column, representing the importance of each physical location interval.

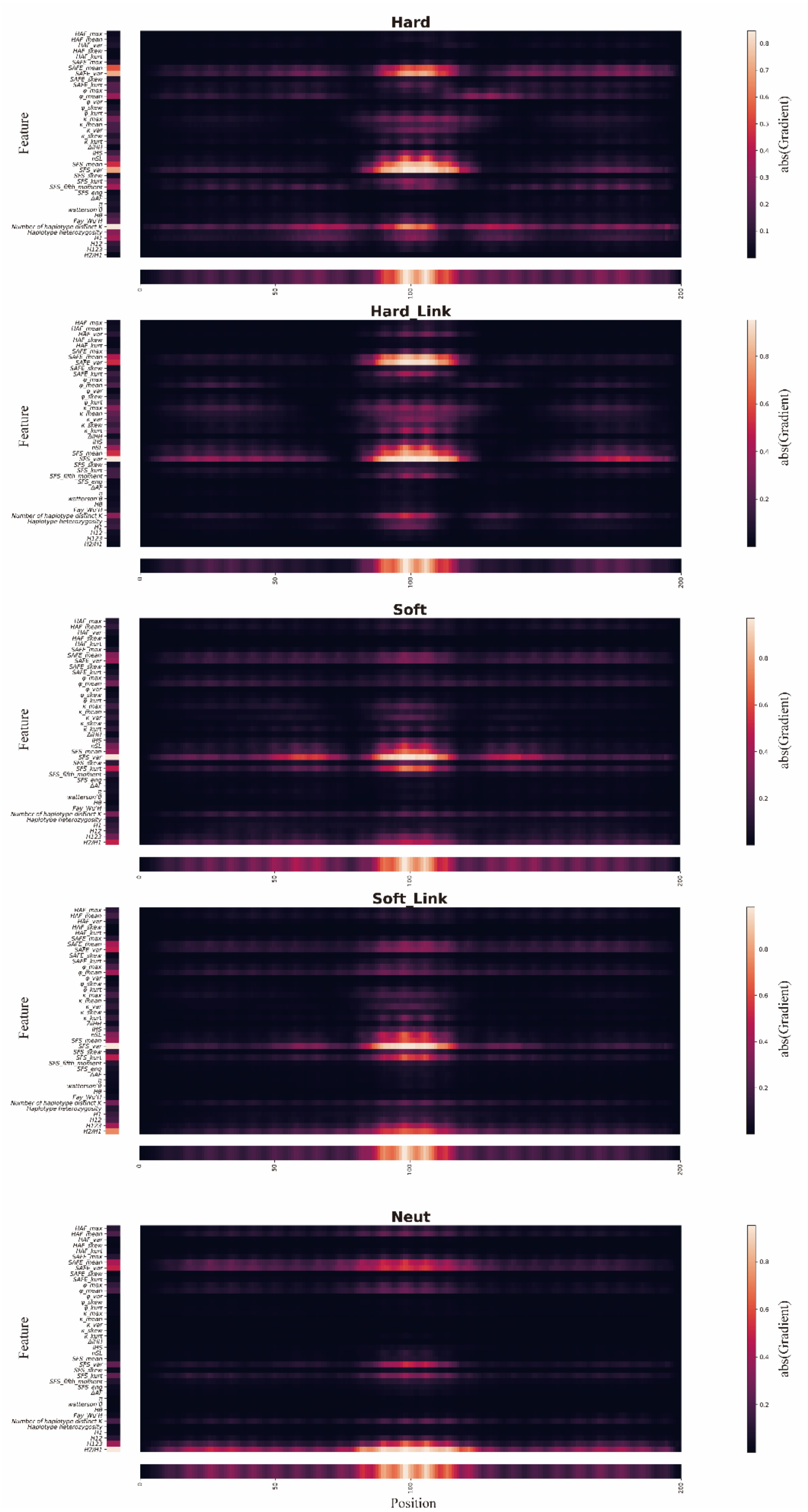

Figure S15 Saliency maps in biased scenario. Legend is same as Figure S14.

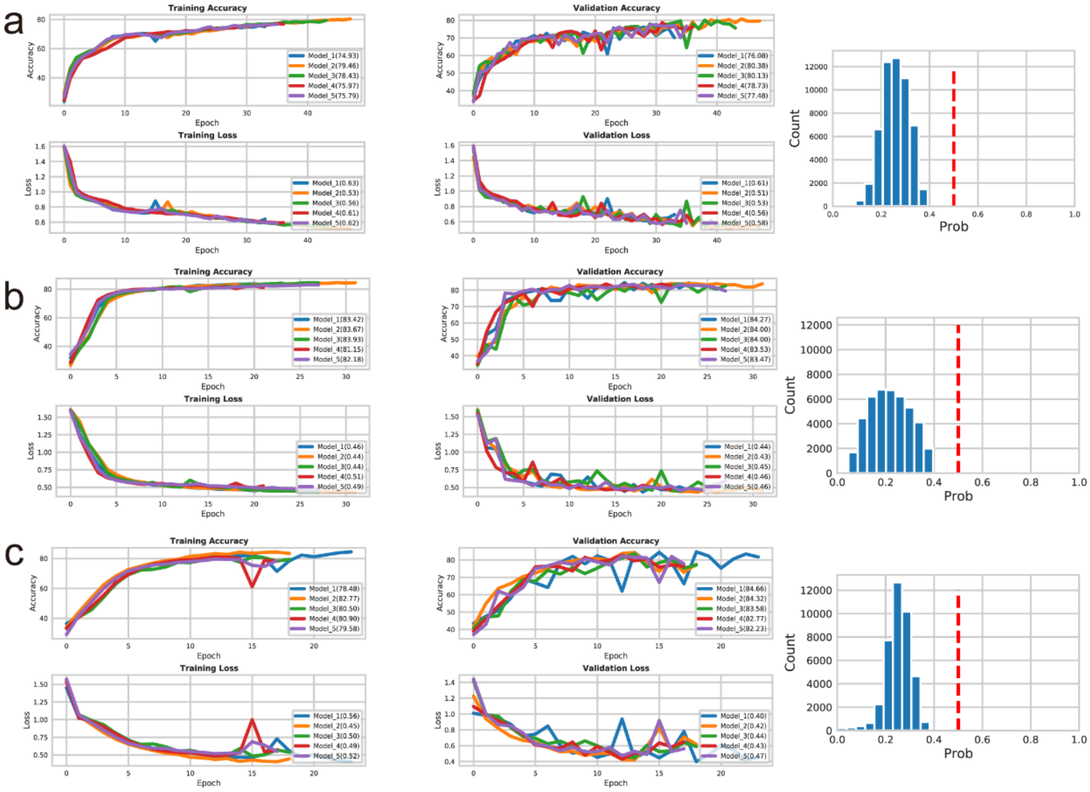

Figure S16 The training process and resolution results of the discriminator for real genome data in three species. The five line-graphs correspond to the training process of the five sub-models, which record the accuracy and loss of the model on the training set and validation set on four sub-figures. The histogram shows the frequency distribution of the discriminator's identification results for real data, where Prob greater than 0.5 (red dotted line) indicates that the model identifies the sample as real data. Figure (a) shows the results for the CEU population, figure (b) shows the results for the domestic pig population, and figure (c) shows the results for the BFS population.

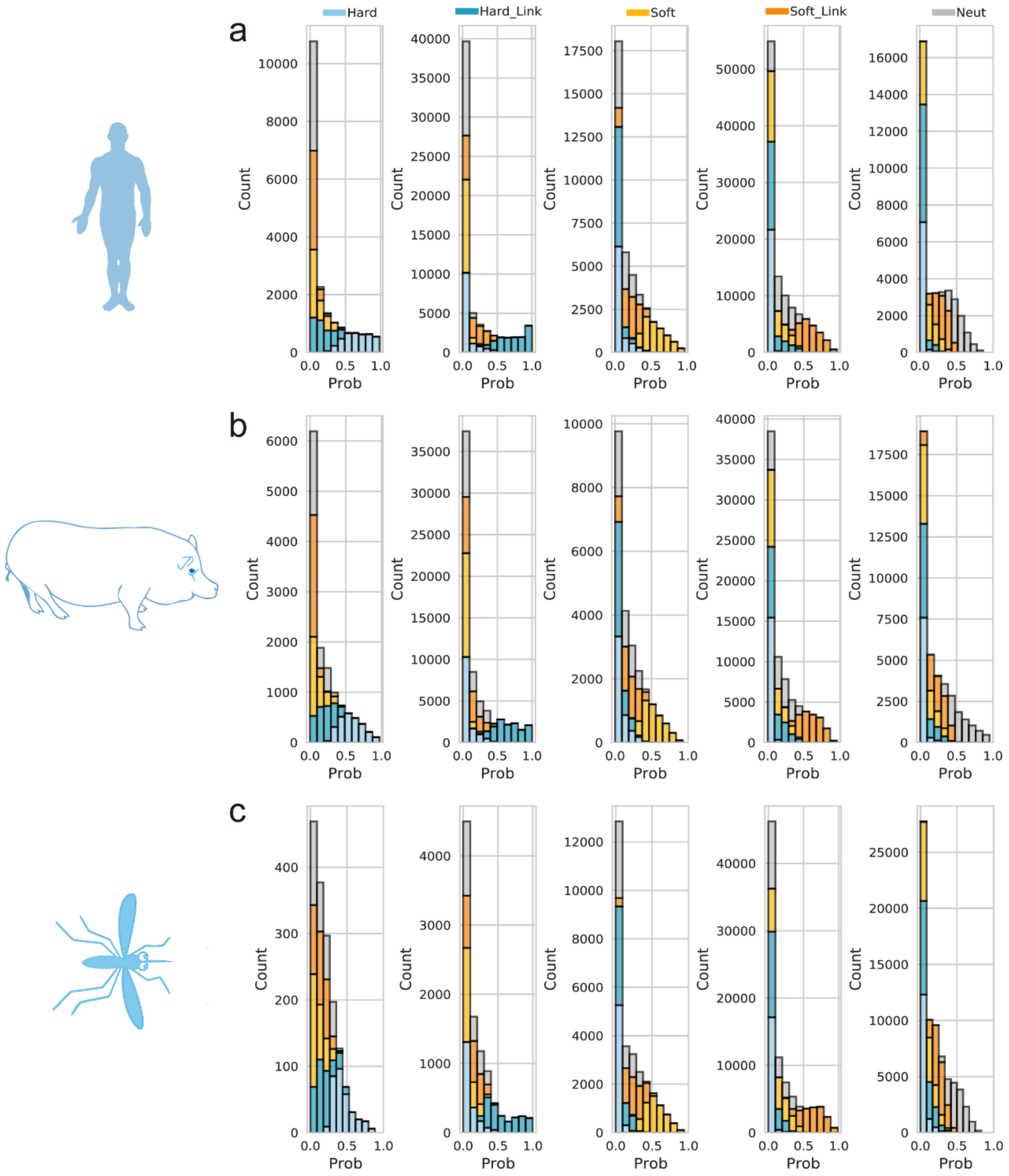

Figure S17 Statistical distributions of predicted results for three species. From left to right, the probability distributions of samples predicted as hard sweep, linked hard sweep, soft sweep, linked soft sweep, and neutral are shown. Light blue represents the probability of samples being predicted as hard sweep, blue represents the probability of samples being linked hard sweep, orange represents the probability of samples being predicted as soft sweep, orange-brown represents the probability of samples being predicted as linked soft sweep, and gray represents the probability of samples being predicted as neutral. Figure (a) shows the five frequency histograms of the CEU

population, figure (b) shows the five frequency histograms of the LW population, and figure (c) shows the five frequency histograms of the BFS population.

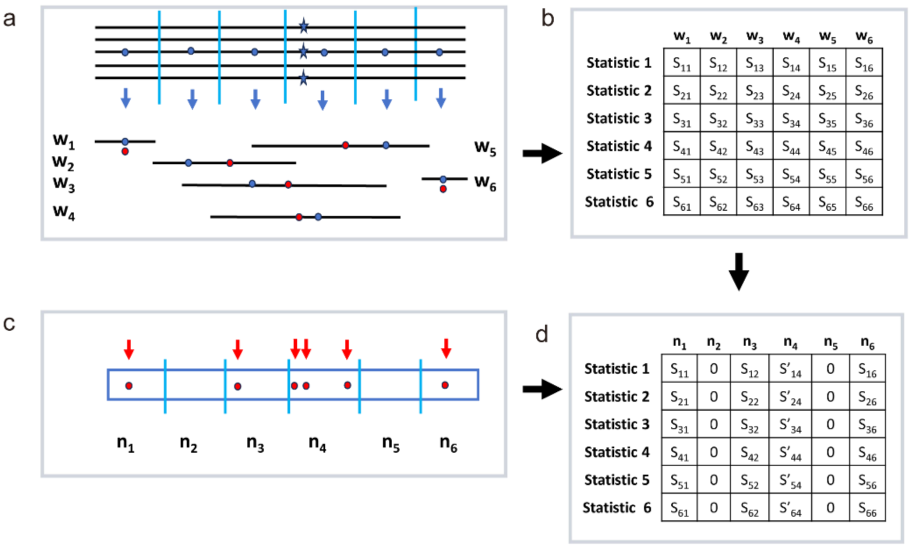

Figure S18 Genome selective sweep feature engineering construction flowchart. Blue stars represent selected site, blue grids indicate central SNPs, and red grids represent the mean position of the SNP windows. (a) illustrates dropping n windows which obtain by dropping n grids and extend k SNPs to each end, and the positional information be encoded in figure (c). (b) shows the computation of 40 summary statistics for n windows, resulting in 40*n matrix in shape. (d) is the calculation of the mean of statistical values based on m physical location interval, and obtain a 40*m matrix in shape.

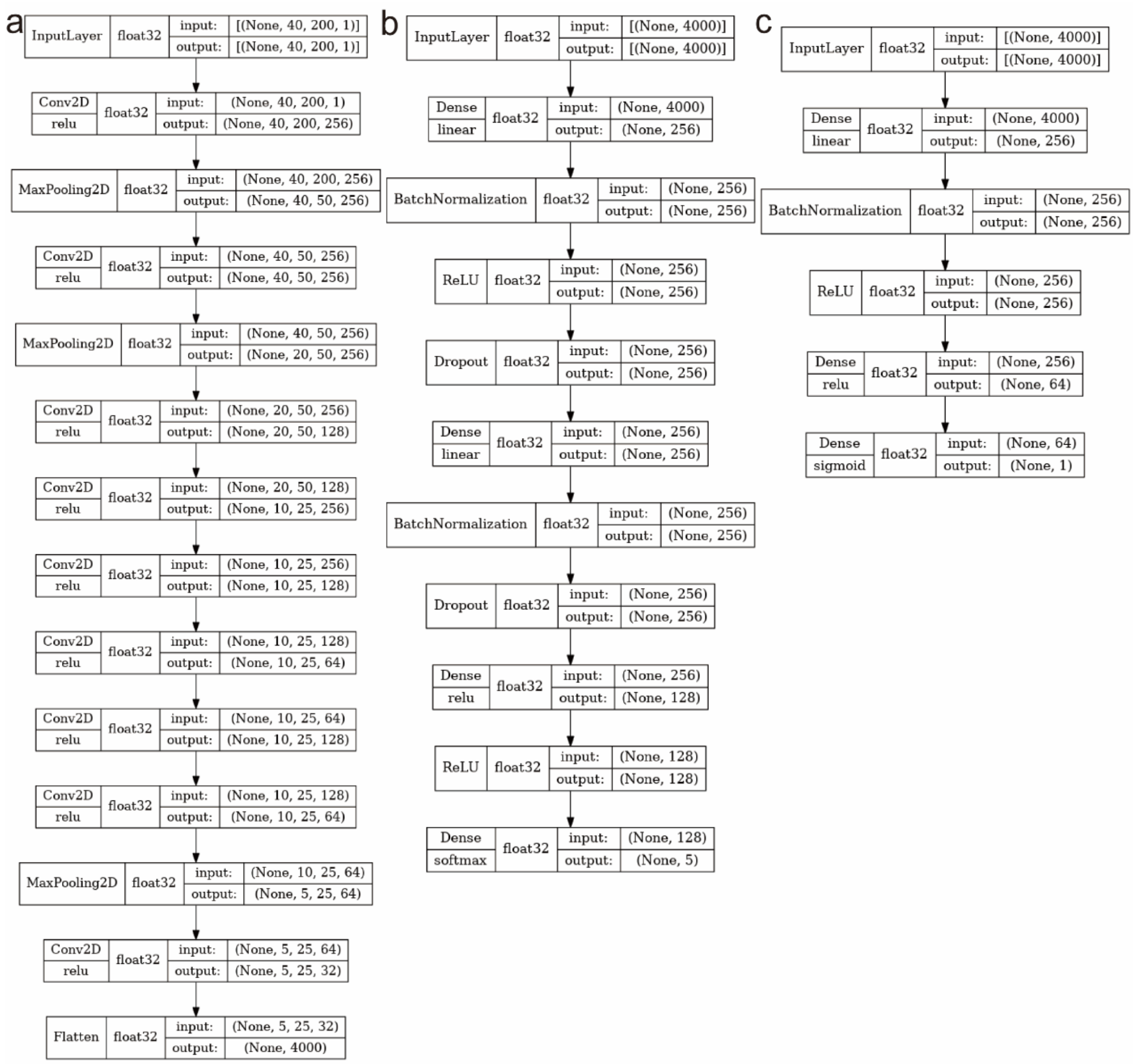

Figure S19 Module structures of DASDC. (a) Structure of feature extractor. This structure uses two stacked convolution layers and max-pooling layers, with kernel sizes of 1*5 and 3*1 for the convolution layers; two bottleneck structures and a max-pooling layer, and finally passes through a convolution layer and flatten layer. (b) Structure of feature classifier. This structure uses two fully connected layers with batch normalization layers and ReLU activation function, one fully connected layer with ReLU activation function, and a fully connected layer with softmax output for predicting the distribution. The connections between the neurons in the fully connected layers use the dropout strategy. (c) Structure of domain discriminator. This structure uses one fully connected layer with a batch normalization layer and ReLU activation function, one fully connected layer with ReLU activation function, and a fully connected layer with sigmoid output for predicting the distribution.

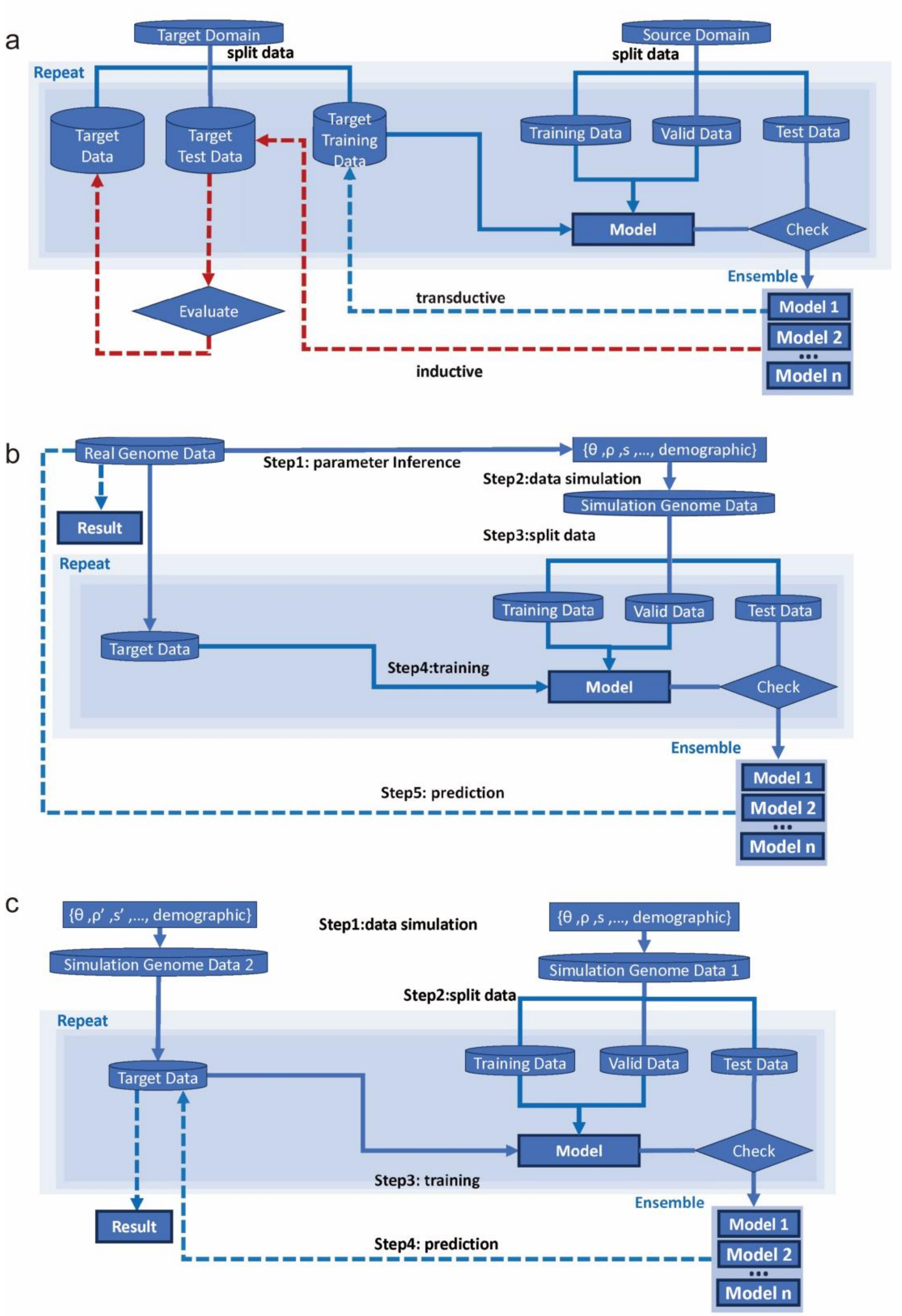

Figure S20 (a) Flowchart of domain adaptation. The blue dotted arrow represents transductive learning, and the red dotted arrow represents inductive learning. (b) Flowchart of domain adaptation used in real genome data. (c) Flowchart of domain adaptation used in two simulation genome data sets with mismatch.

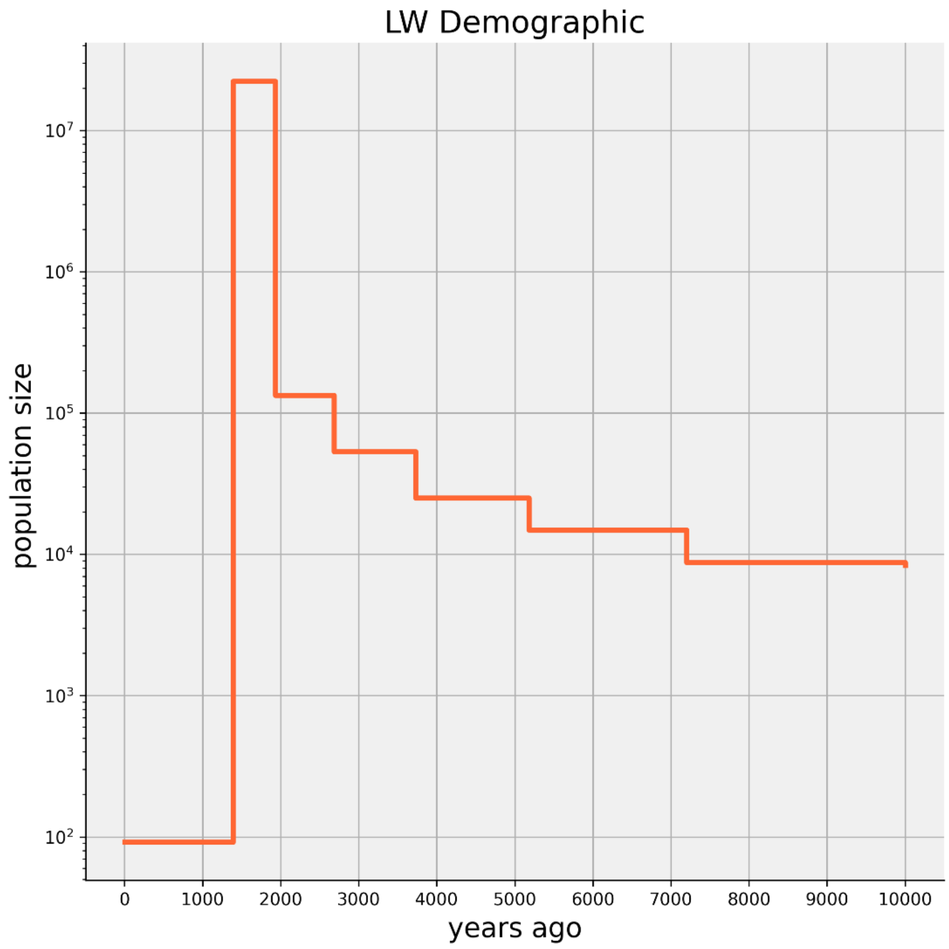

Figure. S21 Demographic inference for large white population by Relate. These figure shows the population size change from 10kys to now. The horizontal axis represents the number of years traced back, and the vertical axis represents the effective population size at the corresponding time.

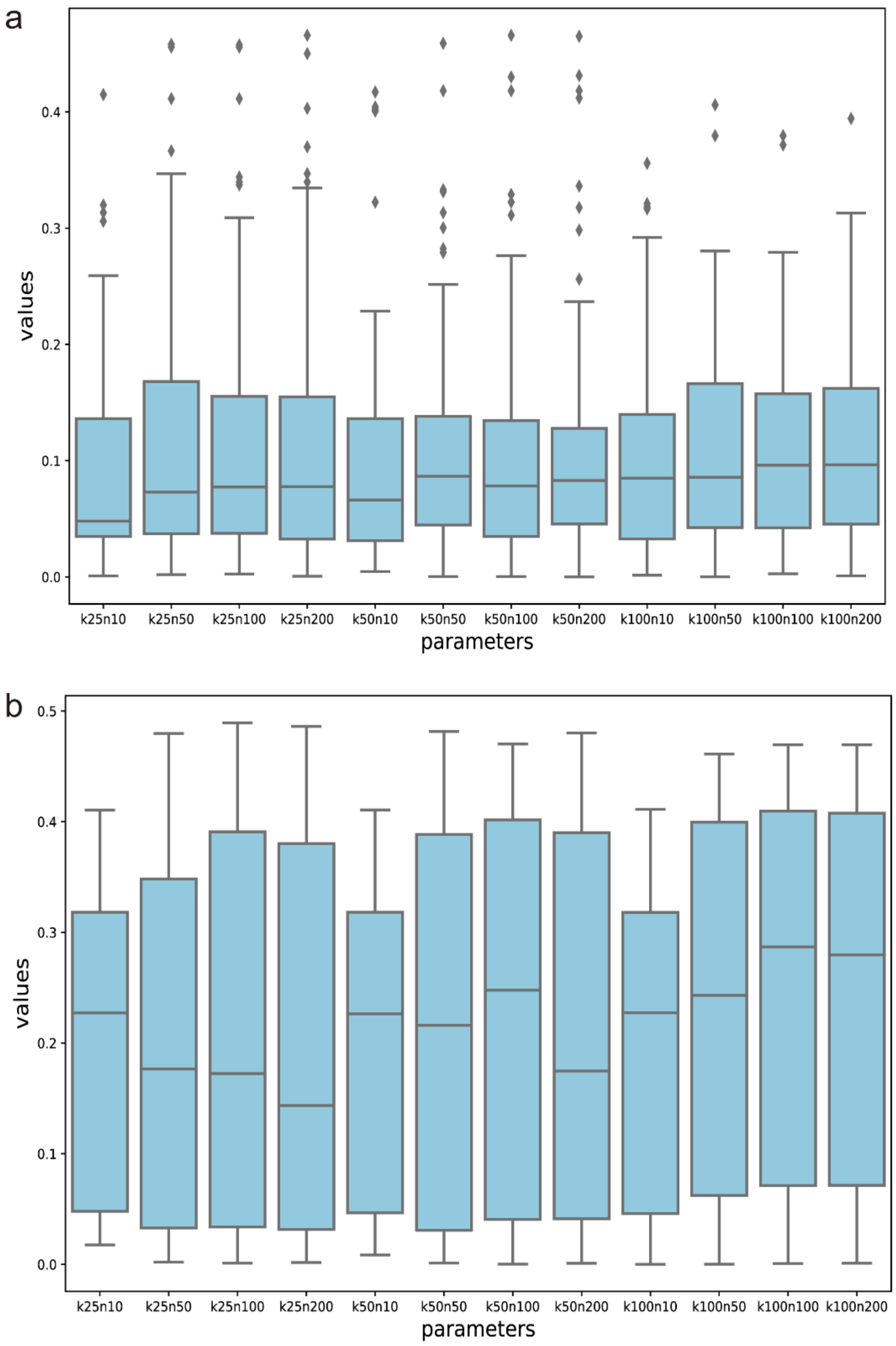

Figure S22 The labels on the x-axis represent different combinations of k and n, The values on y-axis represent the relative distance of selected site with the minimum of nucleotide diversity π (relative distance = distance / L, L is the length of the segment). (a) Evaluated using 1000 instances of hard sweeps. (b) Evaluated using 1000 instances of soft sweeps.

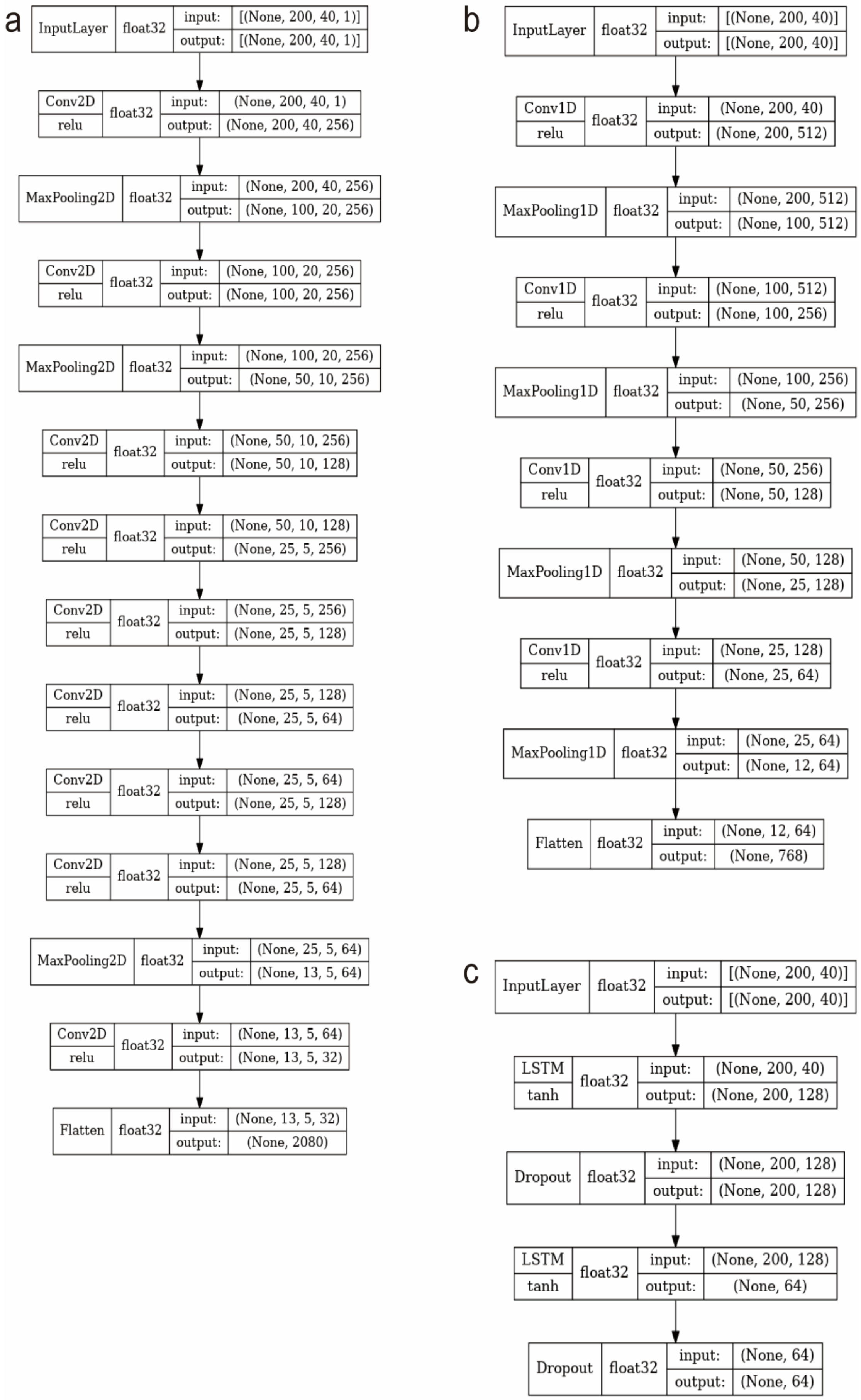
a
InputLayer | float32 | input: [(None, 200, 40, 1)] | output: [(None, 200, 40, 1)]
Conv2D relu | float32 | input: (None, 200, 40, 1) | output: (None, 200, 40, 256)
MaxPooling2D | float32 | input: (None, 200, 40, 256) | output: (None, 100, 20, 256)
Conv2D relu | float32 | input: (None, 100, 20, 256) | output: (None, 100, 20, 256)
MaxPooling2D | float32 | input: (None, 100, 20, 256) | output: (None, 50, 10, 256)
Conv2D relu | float32 | input: (None, 50, 10, 256) | output: (None, 50, 10, 128)
Conv2D relu | float32 | input: (None, 50, 10, 128) | output: (None, 25, 5, 256)
Conv2D relu | float32 | input: (None, 25, 5, 256) | output: (None, 25, 5, 128)
Conv2D relu | float32 | input: (None, 25, 5, 128) | output: (None, 25, 5, 64)
Conv2D relu | float32 | input: (None, 25, 5, 64) | output: (None, 25, 5, 128)
Conv2D relu | float32 | input: (None, 25, 5, 128) | output: (None, 25, 5, 64)
MaxPooling2D | float32 | input: (None, 25, 5, 64) | output: (None, 13, 5, 64)
Conv2D relu | float32 | input: (None, 13, 5, 64) | output: (None, 13, 5, 32)
Flatten | float32 | input: (None, 13, 5, 32) | output: (None, 2080)
b
InputLayer | float32 | input: [(None, 200, 40)] | output: [(None, 200, 40)]
Conv1D relu | float32 | input: (None, 200, 40) | output: (None, 200, 512)
MaxPooling1D | float32 | input: (None, 200, 512) | output: (None, 100, 512)
Conv1D relu | float32 | input: (None, 100, 512) | output: (None, 100, 256)
MaxPooling1D | float32 | input: (None, 100, 256) | output: (None, 50, 256)
Conv1D relu | float32 | input: (None, 50, 256) | output: (None, 50, 128)
MaxPooling1D | float32 | input: (None, 50, 128) | output: (None, 25, 128)
Conv1D relu | float32 | input: (None, 25, 128) | output: (None, 25, 64)
MaxPooling1D | float32 | input: (None, 25, 64) | output: (None, 12, 64)
Flatten | float32 | input: (None, 12, 64) | output: (None, 768)
c
InputLayer | float32 | input: [(None, 200, 40)] | output: [(None, 200, 40)]
LSTM tanh | float32 | input: (None, 200, 40) | output: (None, 200, 128)
Dropout | float32 | input: (None, 200, 128) | output: (None, 200, 128)
LSTM tanh | float32 | input: (None, 200, 128) | output: (None, 64)
Dropout | float32 | input: (None, 64) | output: (None, 64)

Figure S23 Schematic diagram of different feature extractor structures used for comparison. (**a**) Schematic diagram of the 2D CNN structure. This structure uses two stacked convolution layers and max-pooling layers, two bottleneck structures and a max-pooling layer, and finally passes through a convolution layer and flatten layer. (**b**) Schematic diagram of the 1D CNN structure. This structure uses four stacked convolution layers and max-pooling layers, and finally passes through an flatten layer. (**c**) Schematic diagram of the RNN structure. This structure uses two LSTM layers and employs a drop-out strategy after the LSTM layers.
